# Innate immunity mediator STING modulates nascent DNA metabolism at stalled forks in human cells

**DOI:** 10.3389/fmolb.2022.1048726

**Published:** 2023-01-12

**Authors:** Pavlo Lazarchuk, Vy N. Nguyen, Salomé Brunon, Maria N. Pavlova, Julia M. Sidorova

**Affiliations:** Department of Laboratory Medicine and Pathology, University of Washington School of Medicine, Seattle, WA, United States

**Keywords:** human, hydroxyurea, innate immunity, nascent DNA, replication, STING

## Abstract

**Background:** The cGAS/STING pathway, part of the innate immune response to foreign DNA, can be activated by cell’s own DNA arising from the processing of the genome, including the degradation of nascent DNA at arrested replication forks, which can be upregulated in cancer cells. Recent evidence raises a possibility that the cGAS/STING pathway may also modulate the very processes that trigger it, e.g., DNA damage repair or processing of stalled forks.

**Methods:** We manipulated STING levels in human cells by depleting or re-expressing it, and assessed the effects of STING on replication using microfluidics-assisted replication track analysis, or maRTA, a DNA fiber assay, as well as immuno-precipitation of nascent DNA, or iPOND. We also assessed STING subcellular distribution and its ability to activate.

**Results:** Depletion of STING suppressed and its re-expression in STING-deficient cancer cells upregulated the degradation of nascent DNA at arrested replication forks. Replication fork arrest was accompanied by the STING pathway activation, and a STING mutant that does not activate the pathway failed to upregulate nascent DNA degradation. cGAS was required for STING’s effect on degradation, but this requirement could be bypassed by treating cells with a STING agonist. Cells expressing inactive STING had a reduced level of RPA on parental and nascent DNA of arrested forks and a reduced CHK1 activation compared to cells with the wild type STING. STING also affected unperturbed fork progression in a subset of cell lines. STING fractionated to the nuclear fractions enriched for structural components of chromatin and nuclear envelope, and furthermore, it associated with the chromatin of arrested replication forks as well as post-replicative chromatin.

**Conclusion:** Our data highlight STING as a determinant of stalled replication fork integrity, thus revealing a novel connection between the replication stress and innate immune responses.

## Introduction

Innate immunity is a universal cell-intrinsic mechanism of protection against bacteria and viruses. The cGAS DNA sensor and the mediator STING are a branch of the innate immunity pathway that responds to foreign DNA in the cytoplasm by inducing pro-inflammatory cytokines and type I interferons (Hopfner and Hornung, 2020). The cGAS/STING cascade is also triggered by the fragments of cells’ own genomic DNA in the cytoplasm (Chen et al., 2016) generated during DNA damage and repair (de Oliveira Mann and Kranzusch, 2017; Harding et al., 2017; Alvarado-Cruz et al., 2021; Guan et al., 2021), or as a consequence of telomere metabolism (Chen et al., 2017; Nassour et al., 2019), cellular aging (Glück et al., 2017; Takahashi et al., 2018; Lan et al., 2019), and other processes. The cGAS/STING pathway activation contributes to sensitization of cancer cells to radio- and chemotherapy (Parkes et al., 2017; Li and Chen, 2018; Chen et al., 2020) also reviewed in (Hayman and Glazer, 2021), and to their proliferative capacity (Ranoa et al., 2018); furthermore, STING or cGAS are often mutated or silenced in cancer cells (Sun et al., 2013; Xia et al., 2016; Deschamps and Kalamvoki, 2017; Song et al., 2017; de Queiroz et al., 2018).

DNA replication can be a source of extra-genomic DNA fragments, in particular if the complex process of the discontinuous DNA synthesis by multiple forks is stressed by genetic deregulation or a genotoxic drug (Técher and Pasero, 2021). Impaired Okazaki fragment processing is an example of the former, and the latter is exemplified by nucleotide insufficiency, often modeled by treatment of cells with ribonucleotide reductase inhibitor hydroxyurea (HU). In both cases the observed consequences include changes in fork progression rates and appearance of nicks and single stranded (ss) gaps in nascent DNA, underlying which are the adaptive responses of fork reversal and nascent DNA degradation (NDD) though unwinding, excision and nucleolytic digestion (Rickman and Smogorzewska, 2019; Thakar and Moldovan, 2021). In cancer cells these responses are often deregulated, which correlates with sensitivity to chemotherapies that interfere with DNA replication (Chaudhuri et al., 2016; Rondinelli et al., 2017; Hill et al., 2018; Cong et al., 2021). One prominent example is the upregulated NDD in BRCA1 or BRCA2-deficient breast and ovarian cancers (referred to as a deficiency in fork protection (Schlacher et al., 2011)). Formation and excision of ssDNA during stressed fork processing and particularly its pathological deregulation in cancer cells is a likely source of immune-stimulating DNA species. Indeed, replication fork arrest by HU has been implicated in generating short extra-genomic DNA species (Yang et al., 2007; Bétous et al., 2013; Shen et al., 2015), and the nascent DNA that is removed from HU-stalled forks *via* excision is detectable in the cytoplasm, correlating with the induction of the cGAS/STING pathway (Bhattacharya et al., 2017; Coquel et al., 2018; Emam et al., 2022). More generally, replication stress has been shown to induce the cGAS/STING signaling (Kreienkamp et al., 2018; Donne et al., 2022).

Recent developments have raised a possibility that the cGAS/STING pathway, rather than being merely a downstream responder to fragmented genomic DNA, may modulate the very processes that trigger it. Several proteins that functionally interact with cGAS/STING are known to be involved both in cytoplasmic DNA sensing and DNA damage responses and signaling: MRE11, DNA-PK, IFI16, SAMHD1, and ISG15 (Schlacher et al., 2011; Ferguson et al., 2012; Kondo et al., 2013; Ying et al., 2016; Morchikh et al., 2017; Chen et al., 2018; Coquel et al., 2018; Burleigh et al., 2020; Raso et al., 2020; Sandy et al., 2020; Ka et al., 2021; Luzwick et al., 2021). Furthermore, a fraction of cGAS resides in the nucleus (Orzalli et al., 2015; Liu et al., 2018; Gentili et al., 2019; Volkman et al., 2019; Bai and Liu, 2022), and cGAS suppresses homologous recombination repair (Liu et al., 2018; Jiang et al., 2019). cGAS also suppresses NDD at stalled forks, apparently in the activation- and STING-independent manner (Chen et al., 2020). To our knowledge, the possibility of a similar involvement of STING has not been explored, although STING too may localize to the nucleus (Malik et al., 2014; Cheradame et al., 2021; Dixon et al., 2021) and can affect DNA repair (Cheradame et al., 2021; Taffoni et al., 2021). Here we asked if the status of STING in cells can have an import on DNA replication. Our results indicate that STING depletion and re-expression respectively suppresses and enhances NDD at stalled forks. We also show that activation of STING is important for its effect on stalled forks, and that cGAS is needed for this activation. Our data are consistent with the localization of a subset of cellular STING to the nuclear envelope, and we can detect STING in the pulldowns of newly replicated and replication-arrested chromatin.

## Results

### STING is found in the nuclear envelope and chromatin fractions

**FIGURE 1:**
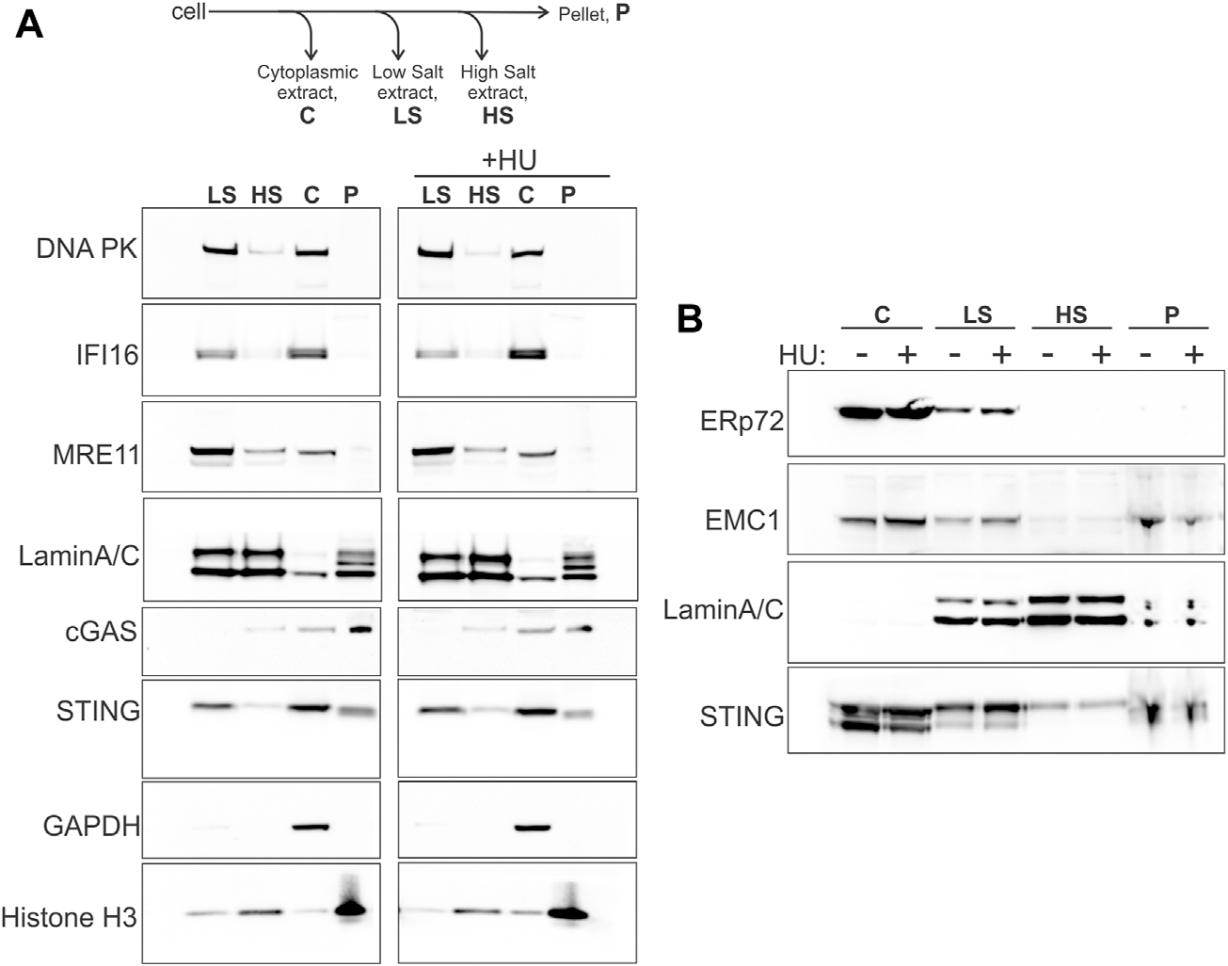
STING fractionates into nuclear and cytoplasmic fractions. **(A)** A fractionation scheme and Western blots probed for the indicated proteins in the fractions derived from the untreated (left panel) and HU-treated (right panel) WI38 hTERT fibroblasts. HU treatment was with 5 mM HU for 5.5 h. **(B)** The same samples as in **(A)** were re-run and probed for the proteins ERp72, EMC1, Lamin A/C, and STING. The loaded amounts of chromatin pellet were increased two-fold compared to **(A)**. A secondary band in the STING blot is STING degradation product. Here and elsewhere Western blot images are boxed to indicate the panels that were independently adjusted for brightness or come from separate protein gels.

STING is an ER protein that is typically seen in the perinuclear membranes upon activation (Ishikawa and Barber, 2008; Ishikawa et al., 2009; Saitoh et al., 2009). However, STING may also be physically present and functionally involved in the nucleus, specifically, in the inner nuclear membrane, INM (Schirmer et al., 2003; Malik et al., 2014; Cheradame et al., 2021; Dixon et al., 2021), enabling a view of STING as a more direct participant in nuclear processes, similar to cGAS (de Oliveira Mann and Hopfner, 2021; Taffoni et al., 2021; Bai and Liu, 2022). We performed serial extraction of nuclei of WI38hTERT fibroblasts and compared STING partitioning to that of cGAS and other known self-DNA sensors—IFI16, DNA-PK, MRE11—that interact with STING and/or are relevant to the cGAS/STING pathway activity as well as function in DNA repair and/or in NDD (Schlacher et al., 2011; Ferguson et al., 2012; Ying et al., 2012; Ying et al., 2016; Morchikh et al., 2017; Coquel et al., 2018; Dunphy et al., 2018; Cheradame et al., 2021; Ka et al., 2021). After separating the cytoplasm (C in Figure 1A) and permeabilizing the nuclei, the latter were extracted with low and high salt buffers (LS and HS in Figure 1A), leaving insoluble pellet (P in Figure 1A), as in (Federation et al., 2020). The high salt fraction is enriched for components of heterochromatin, and the pellet—for tightly bound structural components of chromatin (e.g., histones) and the nuclear envelope, NE (i.e., lamina, nuclear pores, nuclear membranes) (Federation et al., 2020). We probed for GAPDH, Lamin A/C, and histone H3 to confirm the overall partitioning of the cytoplasmic and nuclear components (Figure 1A). As expected, MRE11 (Kondo et al., 2013), DNA-PK (Burleigh et al., 2020) and IFI16 (Iqbal et al., 2016) were present both in cytoplasmic and nuclear extracts, and were efficiently extracted from chromatin in low salt buffer. In contrast, cGAS predominantly segregated with cytoplasm (C) and pellet (P), consistent with the reports of a pool of cGAS ubiquitously associated with chromatin (Jiang et al., 2019; Volkman et al., 2019; Chen et al., 2020). Remarkably, we detected STING in the cytoplasm, low salt, pellet, and high salt fractions (C, LS, P, HS), in this order of abundance. The ER lumen protein ERp72 partitioned to the C, predominantly, and, to a small degree, LS fractions in these same samples (Figure 1B), while the ER transmembrane protein EMC1 that is also found in the NE (Cheng et al., 2019), distributed very similarly to STING in our fractionation scheme (Figure 1B). The presence of the ER lumen marker ERp72 together with the ER membrane marker EMC1 in the C and LS fractions argues partitioning of the peripheral ER to these fractions (Cheng et al., 2019), and suggests that STING of the P and HS fractions is unlikely to come from the peripheral ER. On the other hand, EMC1 distribution in and of itself does not rule out the localization of STING of the P and HS fractions both to the perinuclear ER and NE, including the INM (Dixon et al., 2021) (also see Discussion), given that ER is contiguous both with the INM and the outer nuclear membrane of NE (Ungricht and Kutay, 2015; De Magistris and Antonin, 2018). To note, the distributions of all the above proteins, including MRE11 and DNA-PK, across fractions remained qualitatively unaffected by the hydroxyurea (HU) treatment that elicits severe replication stress by arresting replication forks that can be subjected to NDD (Figure 1A).

### Fork progression rate and nascent strand degradation at stalled replication forks are suppressed by depletion of STING

**FIGURE 2:**
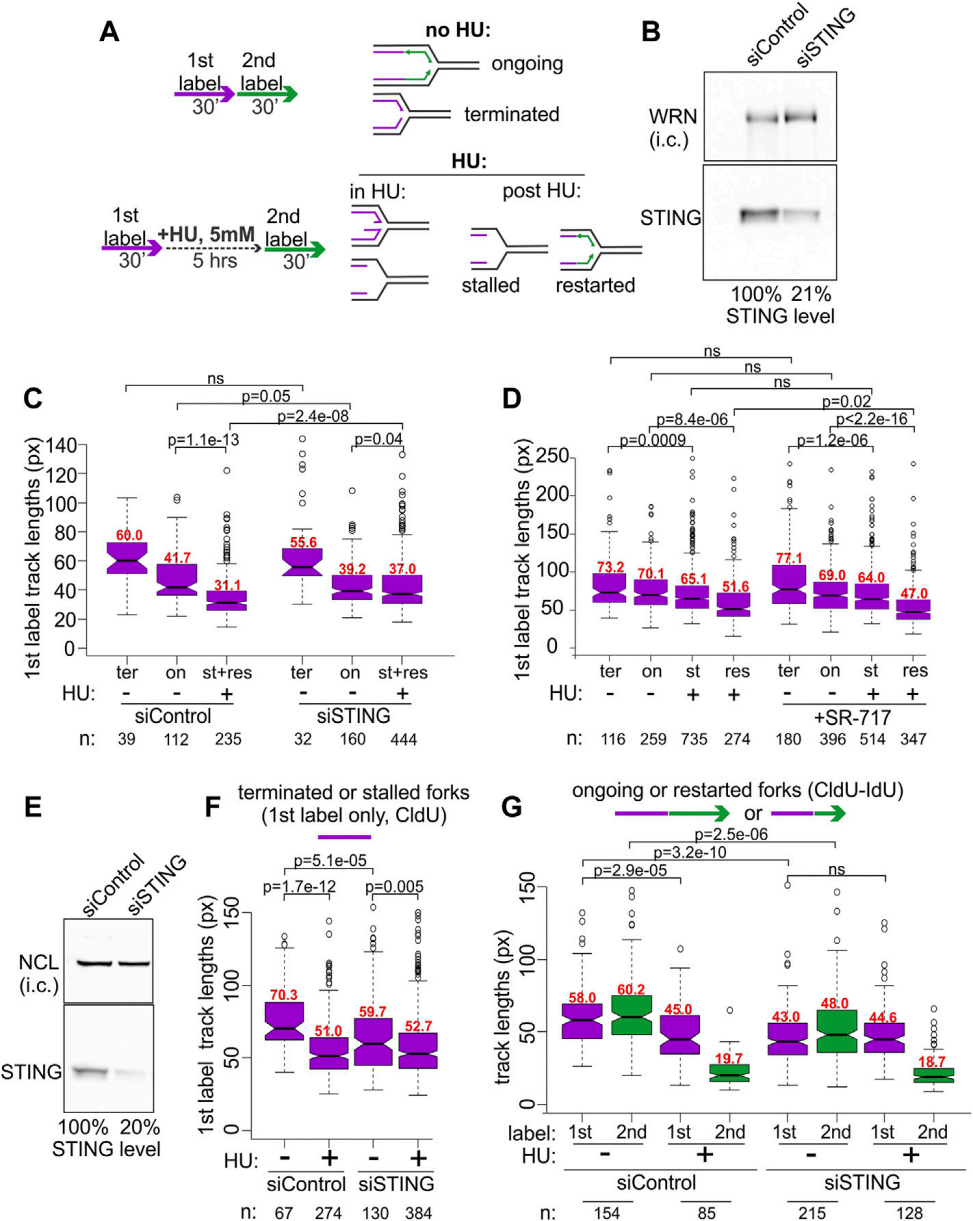
STING depletion affects replication forks. **(A)** Experimental design for detection of NDD (left) and the categories of forks scored in untreated and HU-treated cells using this design (right). **(B)** A Western blot of siRNA-mediated depletion of STING in BRCA1-deficient ovarian cancer line UWB1.289. i.c., internal control. In his case, the WRN protein was used for i.c. **(C)** A boxplot of first label (IdU) track length distributions in terminated (ter) and ongoing (on) forks without HU, and in stalled (st) or restarted (res) forks after HU. The latter two categories were combined and plotted together since restarted forks were extremely rare at 30 min after HU in UWB1.289. **(D)** A boxplot of first label (CldU) track lengths in the terminated, ongoing, stalled, or restarted forks in UWB1.289. Cells were treated the same way as in **(C)** except SR-717 was added at 8 μM during the HU arrest in HU samples or for 5 h before the labeling in no-HU samples. All labeling times were 40 min. The results in **(C,D)** represent two independent experiments each. **(E)** A Western blot of siRNA-mediated depletion of STING in the SV40-transformed fibroblast line GM639. NCL, nucleolin, was used as i.c. **(F)** A boxplot of replication track length distributions of stalled or terminated forks in GM639 transfected with non-targeting and STING siRNAs. **(G)** A boxplot of replication track length distributions of ongoing or restarted forks in GM639 transfected with non-targeting and STING siRNAs. **(F,G)** represent two independent experiments. Here and elsewhere the track length measurements are in pixels (px on Y axes), and 1 pixel approximately equals .62 Kb. *p* values are derived in pairwise KS tests. Numbers of tracks (n) measured in each category are indicated beneath the graphs and median values (in red) of each distribution are shown above the boxes.

BRCA1-deficient breast and ovarian cancer cells display prominent NDD upon replication arrest by HU (Schlacher et al., 2012). This phenotype can be revealed by DNA fiber assays (Quinet et al., 2017), including our microfluidics-assisted replication track analysis, maRTA (Sidorova et al., 2009). One approach is to pulse-label cells with two nucleoside analogs either consecutively (no-HU sample) or with the labels separated by a 5–6 h interval of treatment with HU (HU sample). In a no-HU sample, tracks of the first label represent naturally occurring fork terminations (ter) if they are by themselves, or ongoing forks (on) if they are contiguous with second label tracks (Figure 2A). In a HU sample, tracks of first label (incorporated prior to HU addition) correspond to natural terminations and HU-mediated fork stalls (ter and st in Figure 2). Tracks of contiguous first and second labels correspond to stalled forks that restarted (res in Figure 2) replication after HU. If NDD occurs during HU arrest, first label tracks lose some of the label, effectively becoming shorter in comparison with the first label tracks of a no-HU sample (Figure 2A). Both the forks that restart replication after HU and those that do not can undergo degradation in HU.

We used siRNA to transiently deplete 80% of STING (Figure 2B) in the BRCA1-negative UWB1.289 ovarian cancer cell line and subjected the depleted cells and controls to the labeling regimen depicted in Figure 2A. The majority of forks of UWB1.289 cells did not restart within the first 30 min after HU, and thus the data for stalled and restarted forks were combined for the analysis (Figure 2C, st+res).

As expected, due to the BRCA1 deficiency first label tracks of the forks that have undergone an HU arrest (lane 3 st+res in Figure 2C) were dramatically shorter compared to first label tracks of the forks that had not been arrested by HU (lanes 1 and 2, ter and on**,** in Figure 2C) in the control cells. Strikingly, in STING-depleted cells the shortening of tracks due to HU arrest was markedly reduced, though not eliminated (Figure 2C, compare for example lanes 5 to 6 vis-à-vis lanes 2 to 3 of the control). Depletion of STING also appeared to reduce the lengths of tracks in a no-HU sample, suggesting a reduced fork progression rate (Figure 2C, compare lanes 1 and 2 to 4 and 5), however, the effect did not reach statistical significance. The effects of STING depletion on fork activity in an ovarian cancer cell line prompted us to ask if overactivation of STING would have an opposite effect on forks. SR-717 was recently identified as a cell-permissible cGAMP mimic STING agonist capable of activating the pathway within hours of addition (Chin et al., 2020). We treated UWB1.289 cells with SR-717 during the 6 h of the HU arrest, or, in the no-HU controls, for 6 h prior to labeling and during the pulse of the first label (Figure 2D). Recovery time after HU was increased to 40 min to enable more stalled forks to restart. SR-717 treatment during HU arrest caused a modest though significant increase in NDD in restarting forks but did not affect fork progression in no-HU samples. These data indicate that activation of STING may be involved in its effect on stalled replication forks.

To further characterize STING effect on replication, we asked if we can reproduce it in a BRCA1-proficient cell line. A derivative of the SV40-transformed fibroblast cell line GM639 that we have previously used in our studies and that carries a wild type BRCA1 and a benign SNP variant of BRCA2, Q2384K (Juarez et al., 2018), displays some NDD upon HU arrest. We depleted STING by siRNA in the GM639 background (Figure 2E). STING depletion reduced fork rates in the absence of HU, as evidenced by shorter tracks in terminated (Figure 2F, compare lanes 1 and 3) and ongoing forks (Figure 2G, compare lanes 1, 2 and 5, 6). At the same time, the shortening of first label tracks caused by HU was once again reduced in STING-depleted GM639 compared to controls with non-targeting siRNA. This reduction was evident both for the forks that did not restart within the first 30 min after HU (Figure 2F, compare lanes 1–2 vs. 3–4) and the forks that restarted (Figure 2F, compare lanes 1–3 vs. 5–7).

Lengths of tracks synthesized by forks restarted after HU arrest are typically much shorter than the tracks synthesized over the same time in untreated cells (Sidorova et al., 2008; Sidorova et al., 2013), and STING depletion did not change this trend (Figure 2F, lanes 4 and 8). Taken together the data suggest that siRNA-mediated depletion of STING can reduce NDD in BRCA1-null and BRCA-proficient backgrounds. STING depletion also showed a tendency to reduce progression of unperturbed forks though the extent was variable between these backgrounds.

### cGAS contributes to the nascent DNA degradation at stalled forks *via* activation of STING

We were interested to explore the contributions of cGAS and of the activation of the cGAS/STING pathway to the replication phenotypes we observed with STING. cGAS deficiency is expected to disrupt the effect of STING on NDD if the latter depends on the activation of STING by cGAS. To test this, we used the cGAS knockout HeLa cells and their derivative harboring the doxycycline-inducible cGAS-GFP, both described previously (Volkman et al., 2019).

**FIGURE 3:**
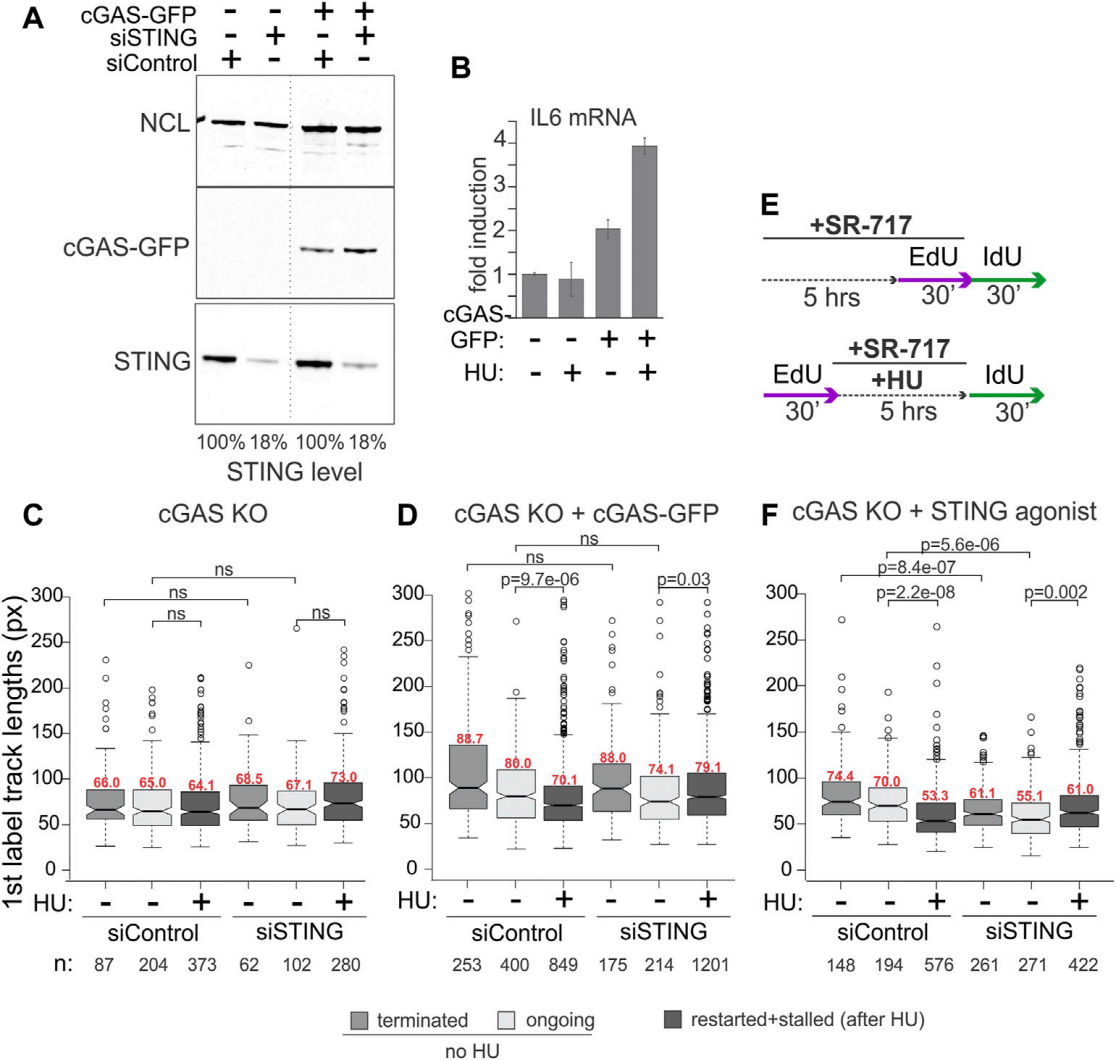
cGAS contributes to the activation of STING, which is required for STING effect on replication forks. **(A)** Depletion of STING and induction of cGAS-GFP expression in the cGAS knockout (KO) HeLa cells. Cells were transfected with the indicated siRNAs and in 24 h doxycycline was added to all cells (KO and complemented) at 400 ng/ml for another 16–24 h. A dotted line marks a spliced lane. **(B)** qPCR measurements of *IL6* mRNA in cGAS KO and cGAS KO/cGAS-GFP HeLa incubated with doxycycline as in **(A)** to induce cGAS-GFP, and treated with 5 mM HU for 5 h. Error bars are standard deviations. **(C,D)** Boxplots of first label (EdU) track length distributions in doxycycline-treated cGAS KO cells **(C)** and cGAS KO + cGAS-GFP cells **(D)**. The experimental design and HU treatment was as in Figure 2, and track length values in restarted and stalled forks after HU are combined due to the low fraction of restarted forks. The results represent two independent experiments. **(E)** A modification to the experimental design to include a STING agonist SR-717 treatment. **(F)** Boxplots of first label (EdU) track length distributions in cGAS KO cells with and without an incubation with SR-717 (4 μM). The results represent two independent experiments. *p* values in **(C–F)** are determined in KS tests.

The STING pathway activation can be gauged by the induction of the pathway’s major transcriptional targets, type I interferons (IFNs) and inflammatory cytokines, e.g., respectively, the IFN beta, *IFNB1*, and interleukin 6, *IL6*, genes (Galluzzi et al., 2018). Overnight incubation with doxycycline induced cGAS-GFP expression (Figure 3A) and conferred HU-inducibility on the expression of *IL6* compared to the parental cGAS KO incubated with doxycycline (Figure 3B), confirming activation of the cGAS/STING pathway by HU. To note, *IFNB1* expression was low and uninducible in these cGAS KO and complemented HeLa cells (not shown), as expected from a prior study (Suter et al., 2021). The ectopic cGAS partitioned similarly to the endogenous cGAS upon fractionation (compare Supplementary Figures S1A, B, Figure 1) and did not affect STING partitioning in a significant or reproducible manner.

For maRTA, the cGAS-GFP-complemented and cGAS KO cells were transfected with control and STING-targeting siRNAs as in Figure 2 and incubated with doxycycline overnight prior to the experiments. Very minor if any shortening of tracks was observed in HU-treated cGAS KO cells, rendering a contribution from STING depletion undetectable (Figure 3C). In contrast, cGAS-GFP expressing cells exhibited measurable shortening of tracks in HU, and depletion of STING suppressed this shortening in a manner similar to its effect in the cGAS-proficient cell lines described earlier (Figure 3D). To test if the role of cGAS in this context was to activate STING, we asked if exposure to a STING agonist would bypass the genetic requirement for cGAS seen in Figures 3C, D. Indeed, treatment of siRNA-transfected cGAS KO cells with SR-717 during the 5-h incubation with HU (Figure 3E) elicited STING-dependent shortening of tracks (Figure 3F). The effect of SR-717 on HU-arrested forks was also reproduced in the cGAS-deficient (de Queiroz et al., 2018) ovarian carcinoma cell line SKOV-3 (Supplementary Figure S1C). SKOV-3 displayed no track shortening in HU, but treatment with SR-717 along with HU induced track shortening (Supplementary Figure S1D). Overall, the data are consistent with the activated STING being a positive regulator of NDD at HU-arrested forks in cancer cells, with cGAS as an upstream activator of STING in this context.

As before, in the absence of HU the effect of STING depletion on track lengths was variable. In cGAS KO HeLa, tracks became shorter upon STING depletion when both control and depleted cells were treated with SR-717 (Figure 3F). This STING-dependent effect was also discernible when cGAS-GFP was expressed, however, it was smaller and did not reach statistical significance (Figure 3D). SR-717 by itself did not change track lengths in SKOV3 (Supplementary Figure S1D). In HeLa cGAS KO, however, it elicited a small increase, statistically significant for ongoing forks only (Supplementary Figure S1E). The effect of STING depletion could not be ascertained in SKOV-3, as we could not deplete STING in these cells to any significant degree (Supplementary Figure S1C). Overall, the data indicate that STING activity may correlate with longer tracks, i.e., faster fork progression, in unperturbed S phase of some but not all backgrounds.

### Reconstitution of activation-competent STING in STING-deficient cancer cells enhances nascent DNA degradation

**FIGURE 4:**
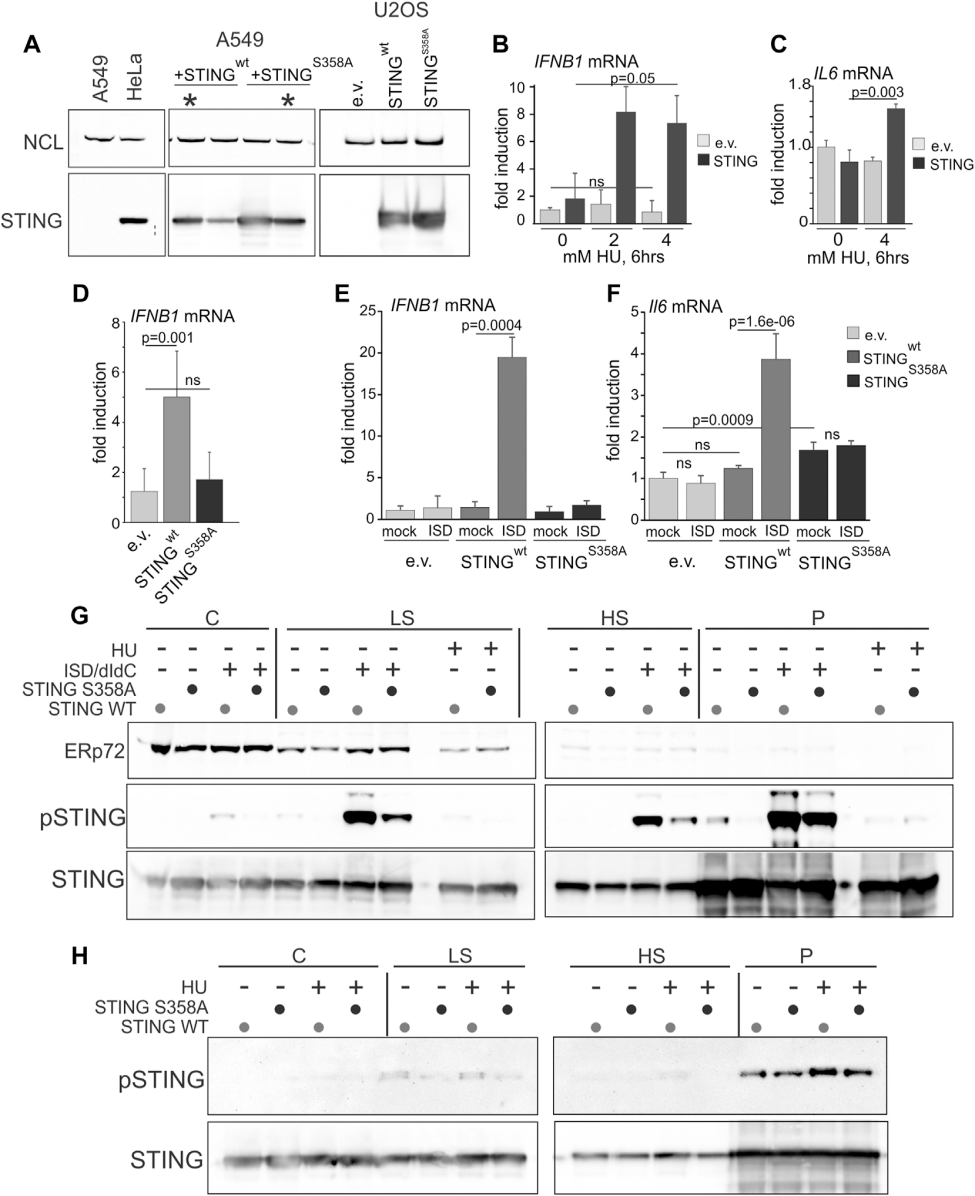
Serine 358 to alanine mutation of STING disrupts its ability to activate and induce downstream transcriptional targets in response to exogenous DNA or replication arrest by HU. **(A)** A Western blot of re-expression of wild type STING, or its S358A mutant in A549 and U2OS. Cells were stably transfected with empty lentiviral vector pTRIP-SFFV-mtagBFP-2A (e.v.) or with the same vector expressing variants of STING (pTRIP-SFFV-mtagBFP-2A STING). Cells were flow-sorted for BFP expression, and several fractions were cultured and analyzed for STING expression to select populations with matching levels of the protein. Examples of the A549 selections are marked by asterisks. **(B)** qPCR measurements of *IFNB1* mRNA induction in U2OS cells expressing STING or empty vector (e.v.) treated with 0, 2 or 4 mM HU for 6 h. **(C)** qPCR measurements of *IL6* mRNA induction in U2OS cells expressing STING or empty vector (e.v.) treated with 0 or 4 mM HU for 6 h. **(D)** qPCR measurements of *IFNB1* mRNA induction in U2OS cells expressing the indicated STING variants or empty vector, incubated with 5 mM HU for 6 h. **(E,F)** qPCR measurements of *IFNB1* **(E)** or *IL6* **(F)** mRNA induction in U2OS cells expressing the indicated STING variants or empty vector, and mock-transfected or transfected with interferon-stimulating DNA (ISD). qPCR results represent two independent experiments each. Error bars throughout are standard deviations. *p* values were calculated on ΔΔCq values in one-tailed two-sample t-tests. The color key [right of **(F)**] is common for **(E,F)**. **(G)** Western blots of the fractionated extracts of U2OS cells expressing STING WT or S358A. Cells were transfected with ISD/poly dI/dC mix or mock-transfected and incubated for 6 h prior to harvest. The HU-treated samples, included for comparison, were incubated with 5 mM HU for 6 h. Blots were probed for ERp72, STING S366P, and STING. **(H)** Western blots of the fractionated extracts of U2OS cells expressing STING WT or S358A and probed for STING S366P and STING. Where indicated, HU treatment was for 5 h s at 5 mM. Note the higher exposure of STING S366P blots compared to those in **(G)** due to a lower S366P signal.

Many cancers have low or absent STING expression due to epigenetic silencing, suggesting that this confers selective advantage in tumorigenesis (Hoong et al., 2020; Hayman et al., 2021). We were interested to test if re-expression of STING in STING-negative cancer cell lines elicited the replication phenotypes opposite to those produced by STING depletion. In addition, to further examine the role of STING activation in its effect on NDD, we re-expressed a STING mutant that has an activation defect. U2OS osteosarcoma cells do not express STING (Deschamps and Kalamvoki, 2017) and are frequently used in replication stress studies, where they display moderate to no NDD (Thangavel et al., 2015). A549 lung carcinoma cells express a negligible amount of STING (Ranoa et al., 2018). We stably re-expressed STING in these cells from an integrated lentiviral construct pTRIP-SFFV-mtagBFP-2A STING (Cerboni et al., 2017) that enables selection of transduced cells by flow-sorting by BFP expression. STING expression was verified in cultured flow-sorted cells (Figure 4A). We also introduced into the ectopic STING a S358A mutation, which eliminates a TBK1 kinase phosphorylation site. This mutation reduces STING aggregation that is part of the activatory response (Li et al., 2015), and reduces binding of the transcription activator IRF3 to STING, which is required for the activatory phosphorylation of IRF3 and downstream signaling (Zhong et al., 2008; Tanaka and Chen, 2012). The STING S358A mutation markedly attenuates the STING pathway activation in response to foreign DNA and viral infection (Zhong et al., 2008; Li et al., 2015; Xie et al., 2018). Ectopic STING S358A was expressed at a level comparable to the wild type protein (Figure 4A), and both wild type and mutant STING had no negative effect on U2OS or A549 cell growth as was evident from serial passaging of these cells.

To verify the functionality of the ectopic STING, we measured induction of the *IFNB1* and *IL6* gene transcripts upon HU arrest. Induction of *IFNB1* and *IL6* mRNA by HU, albeit lower than that achieved upon transfection of foreign DNA (e.g., interferon-stimulating DNA, ISD), was dependent on STING (Figures 4B, C). The STING S358A mutant failed to induce *IFNB1* mRNA in HU (Figure 4D) and did not support *IFNB1* or *IL6* mRNA activation upon transfection of ISD, confirming a defect in its ability to activate its downstream signaling cascade (Figures 4E, F). Interestingly, STING S358A also increased the basal level of *IL-6* mRNA (Figure 4F). Fractionation of cells after transfection of DNA (an ISD/poly dI:dC mixture) and Western blotting showed robust S366 phosphorylation of the wild type STING in low salt (LS), high salt (HS), and pellet (P) fractions, and a significantly reduced phosphorylation of STING S358A throughout (Figure 4G). By comparison, S366 phosphorylation in HU-treated samples was substantially lower than in DNA-transfected samples (Figure 4G), consistent with the RT qPCR results of Figures 4B, C. Nevertheless, comparison of HU-treated and untreated P and HS samples revealed a modest induction of S366 phosphorylation of the wild type STING (Figure 4H, note a higher exposure to reveal the S366P signal). The results confirm the expected defect of STING S358A, and indicate that NE/chromatin fractions of STING (HS and P) can be activated to a similar or higher level compared to the fractions that contains most of the peripheral ER (C and LS).

**FIGURE 5:**
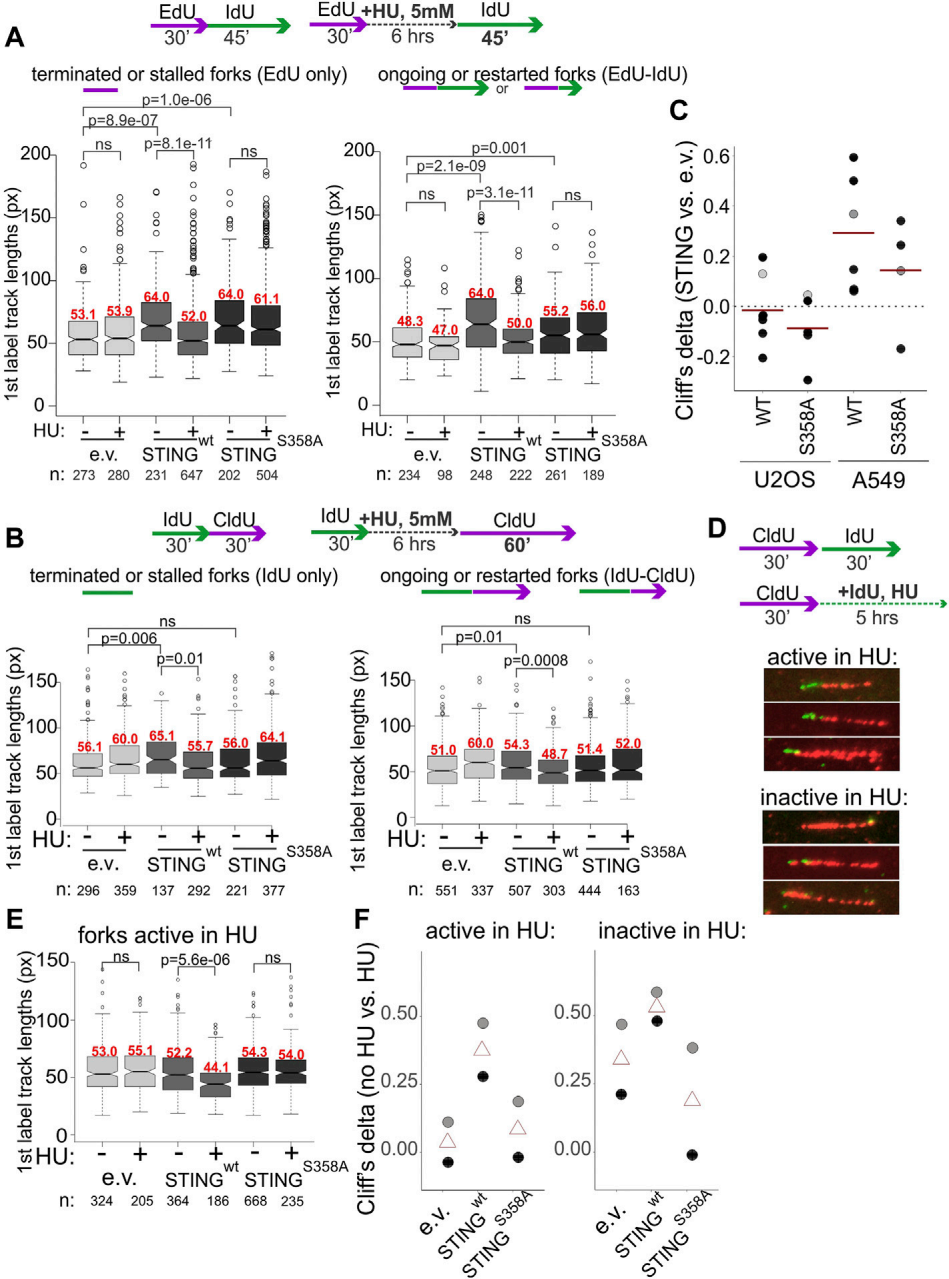
Activation of STING is important for its effect on degradation of nascent DNA. **(A,B)** Experimental design and boxplots of first label (IdU) track length distributions in terminated/stalled forks (left panels), or ongoing/restarted forks (right panels) in the A549 cells **(A)** and U2OS cells **(B)** expressing the indicated transgenes. The results represent two independent experiments each. *p* values are determined in KS tests. Median values of distributions are shown in red above the boxes. **(C)** Cliff’s delta values for the size of differences in unperturbed fork progression between empty vector and STING-expressing cells were calculated from six independent experiments each performed in U2OS and A549 cells. First label track lengths in ongoing forks were used for the calculation, and positive values correspond to longer tracks/faster fork progression in STING-expressing cells compared to the empty vector controls. Red lines are means. Gray circles identify the Cliff’s delta values derived from the experiments shown in **(A,B)**. **(D)** A labeling scheme and examples of forks that incorporate trace IdU (are “active”) or do not incorporate any IdU (are “inactive”) in the presence of 5 mM HU. **(E)** A boxplot of first label (CldU) track length distributions in the “active in HU” forks in U2OS expressing the indicated transgenes. The results represent two independent experiments. *p* values were determined in KS tests. **(F)** Differences between first label (CldU) track length distributions in untreated vs. HU-treated cells with the indicated transgenes were quantified by calculating the respective Cliff’s delta values. Positive values correspond to longer tracks in untreated cells compared to HU-treated cells. Cliff’s delta values from two independent experiments (black and gray circles respectively) and their averages (triangles) were plotted.

We next performed mRTA measurements of fork progression and NDD at HU-stalled forks using variants of the experimental design shown in Figure 2. To increase the fraction of restarting forks, we increased the duration of the second labeling as indicated in Figure 5. STING-deficient controls displayed no shortening of replication tracks at HU-stalled forks both in A549 and U2OS, whereas in cells expressing the wild type STING these tracks became shorter (Figures 5A, B). In contrast, the track length phenotype of the STING S358A mutant was similar to the STING-deficient control (Figures 5A, B). Interestingly, A549 cells had a fairly pronounced phenotype of longer tracks in no-HU samples compared to U2OS cells where this STING effect was minor. This tendency could be traced through multiple independent experiments, as revealed by calculating Cliff’s deltas of track length differences between empty vector on the one hand and STING wild type or mutant samples on the other (Figure 5C). Cliff’s delta is a metric for the size of a difference between two distributions (see Materials and Methods statistical analysis section for more detail), and differences that are not statistically significant typically fall within a −.1 to .1 range. The comparisons in Figure 5C indicate that on average, the increase of track lengths in unperturbed S phase of STING-expressing cells is significant and reproducible only in A549 but not in U2OS, suggesting a STING-dependent increase in fork progression rate in the former. These data also revealed that the mutant STING generally had a statistically significant but smaller effect on unperturbed forks in A549 cells.

The STING re-expression and depletion datasets for which the requisite maRTA data were available, were subsequently compiled to determine whether STING depletion or re-expression affected stability of forks in unperturbed S phase. This included metrics such as percentage of ongoing forks (Supplementary Figure S2A) and asymmetry of diverging forks (Supplementary Figure S2B). To calculate the latter metric, we applied the criteria similar to those in (González Besteiro et al., 2019; Calzetta et al., 2021). In aggregate, the data indicate that manipulation of STING did not result in notable changes in either of these parameters. In addition, efficiency of fork restart after HU was calculated from multiple experiments and was also found unaffected by STING manipulation (Supplementary Figure S2C).

We next used the U2OS model to focus on the phenotype of NDD since it was consistently present in all studied cell lines and was shown by more than one line of experiments to require the cGAS/STING pathway activation and the activation-competent STING. Measurements of S phase fraction, EdU incorporation level, and γH2AX induction upon HU treatment performed in U2OS cells uncovered only very modest differences limited to STING S358A cells (Supplementary Figures S3A–C). HU treatment did not alter partitioning of the wild type or mutant STING between cytoplasmic and chromatin fractions, as we observed for WI38hTERT cells, and the S358A mutation did not disrupt STING distribution to any significant degree (Figure 4G, Supplementary Figure S3D). cGAS distributed among cytoplasmic, high salt and pellet fractions as seen previously, and was qualitatively unaffected by STING status (Supplementary Figure S3D). Treatment with the MRE11 exonuclease inhibitor mirin during and after HU arrest reduced track shortening, confirming that exonucleolytic degradation of nascent DNA was a contributor to this phenotype in U2OS (Supplementary Figure S3E).

In order to distinguish between single forks and two or more converging/diverging forks regardless of whether they are able to restart after HU or not, and to visualize the residual fork activity in HU, we modified the labeling scheme by adding the second label during the HU arrest rather than after it (Figure 5D) as in (Lemacon et al., 2017; Byrum et al., 2019). We measured lengths of first label tracks of two categories: tracks corresponding to single forks that incorporated at least a minimal measurable length of the second label, approx. 15 Kb and tracks that showed no second label incorporation. These categories were named, respectively, “active in HU” and “inactive in HU” for identification purposes only. STING presence correlated with a significant shortening of tracks in HU compared to the control for both categories, albeit the effect was more dramatic for “active in HU” forks (Figure 5E). Cliff’s delta values of the differences between no-HU and HU track length distributions for all three cell lines (Figure 5F) showed similar tendencies in two independent experiments and importantly, that STING S358A was very similar to the control when the “active in HU” forks were measured, and better than the control in suppressing the shortening of tracks in the “inactive in HU” forks, which was a potential gain-of-function phenotype (Figure 5F).

Together, the results further support a connection between STING activity and NDD. They also rule out that the observed effects are due to an increase in nascent DNA breakage either *in vivo* or during processing, since expression of the wild type STING does not increase γH2AX level (Supplementary Figure S3C), and its effect is clearly observed in forks that are captured by the assay as showing activity during HU arrest.

### STING affects the level of RPA at stalled forks, and is found associated with replicating chromatin

STING signaling in response to certain drugs was found to induce homologous recombination deficiency (HRD) through transcriptional inhibition of HR genes, in particular FANCD2 (McLaughlin et al., 2020). FANCD2 protects forks from NDD (Schlacher et al., 2012). However, FANCD2 levels in the STING-deficient and proficient U2OS were not reduced, suggesting that this mechanism may not be engaged under our conditions (Supplementary Figure S3F). To begin to understand what molecular changes may accompany or mediate STING’s effect on stalled forks, we turned to the ssDNA-binding protein RPA. RPA not only highlights the level of ssDNA at forks and thus can inform about the fork state, but also regulates NDD at more than one step (Bhat and Cortez, 2018; Soniat et al., 2019; Berti et al., 2020; Duan and Pathania, 2020). Total RPA levels assayed by Western blotting were similar between the STING-deficient, STING WT, and STING S358A U2OS (Supplementary Figures S4A, B). In addition, there were no consistent differences between the total nuclear RPA levels in STING WT, and STING S358A U2OS as assayed by immunofluorescence (IF) *in situ* (Supplementary Figures S4C, D). However, when IF *in situ* was performed after detergent extraction of cells, which enriched for chromatin-bound RPA, we observed a reduction in the RPA level in STING S358A cells, most prominently after HU treatment (Supplementary Figure S4E).

**FIGURE 6:**
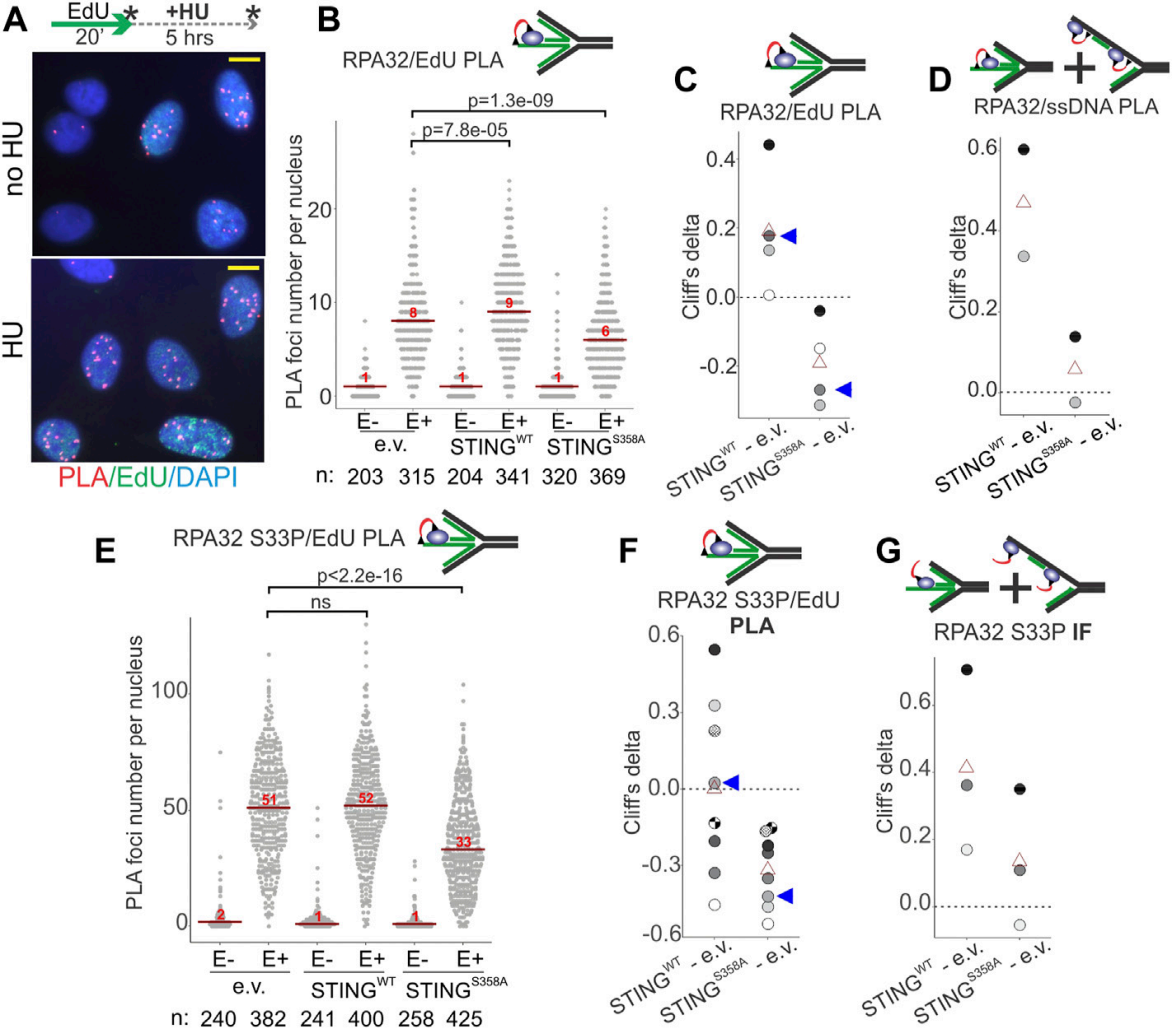
STING affects the levels of RPA on parental and nascent DNA in HU-treated cells. **(A)** Examples of RPA32 to EdU PLA fluorescence in the control U2OS cells, treated as shown. Cells were labeled with EdU and harvested immediately or after a 5 h HU arrest, as shown by asterisks. Scale bar = 10 μm. **(B)** A schematic of the likely substrate for the RPA32/EdU PLA (red arc represents fluorescent signal) and representative distributions of RPA32/EdU PLA foci numbers in the U2OS cells with empty vector (e.v.) or the indicated STING transgenes. Cells were labeled as in **(A)** and EdU was Clicked to a mixture of biotin and Alexa488 azides at a molar ratio of 50:1 to enable simultaneous visualization of EdU-positive cells and PLA signals. PLA foci numbers are shown separately for EdU-negative (E−) and EdU-positive (E+) cells. Red lines are medians and their values are shown above the lines. *p* values were calculated in Wilcoxon tests. **(C)** Magnitudes of differences between RPA32/EdU PLA foci distributions in STING WT vs. control and STING S358A vs. control were calculated as Cliff’s delta values and plotted. Shown are Cliff’s delta values for each of four independent experiments performed and quantified as in **(B)**. Each experiment is identifiable by fill tone of the circle symbols. Red triangles are means. Blue arrowheads identify the values derived from the experiment shown in **(B)**. **(D)** Cliff’s delta values for two biological replicates of an experiment performed as in **(B)** and measuring RPA32/ssDNA PLA foci numbers. Likely substrates for the RPA32/ssDNA PLA are shown above the plot. **(E)** Distributions of RPA32 S33P/EdU PLA foci numbers in the U2OS cells with empty vector or the indicated STING transgenes. Cells were labeled with EdU and treated with HU as in **(A)**. All samples are HU-treated. PLA foci numbers are shown separately for EdU-negative (E−) and EdU-positive (E+) cells. Red lines are medians and their values are shown above the lines. *p* values were calculated in Wilcoxon tests. **(F)** Cliff’s delta values calculated for RPA32 S33P/EdU PLA foci distributions in STING WT vs. control and STING S358A vs. control pairs in each of eight independent experiments performed and quantified as in **(E)** and identifiable by fill tone or fill pattern of the circle symbols. Triangle symbols are means. Blue arrowheads identify the values from the experiment shown in **(E)**. **(G)** Cliff’s delta values calculated for differences of distributions of mean fluorescence intensities (MFI) per nucleus measured by IF *in situ* for RPA32 S33P in STING WT vs. control and STING S358A vs. control cells. MFI values were measured in EdU-positive, HU-arrested cells in three independent experiments. For all Cliff’s delta values, positive values mean that the values of the first distribution in the comparison (e.g., STING WT in the STING WT—e.v. comparison) are overall higher than those of the second distribution, and negative values mean that the reverse is true. Distribution medians are displayed as in **(E)**.

We next used Proximity Ligation Assay (PLA) in addition to IF *in situ* to quantify RPA on nascent and parental ssDNA in HU-treated S phase U2OS cells in more detail. Cells were pulse-labeled with EdU and harvested immediately, or after an HU arrest. As mentioned above, all three cell lines incorporated comparable amounts of EdU per pulse (Supplementary Figure S3B), enabling comparisons of the levels of RPA bound to EdU+ DNA by PLA. PLA was performed with antibodies against biotin (recognizing EdU-biotin conjugates) and the C terminus of the RPA subunit RPA32 (Figure 6A, Supplementary Figure S4F) according to our protocols (Lazarchuk et al., 2019; Lazarchuk et al., 2020). This PLA signal was specific to EdU-positive cells (Supplementary Figure S4F, Figure 6B) and detectable with and without HU (Figure 6A). Remarkably, the HU-arrested wild type STING-expressing U2OS had a higher RPA32/EdU PLA signal than the STING-deficient control (Figure 6B). In contrast, the STING S358A-expressing cells displayed less RPA32/EdU than the control (Figure 6B). These results translated into a highly reproducible differential between the wild type and mutant STING-expressing cells illustrated by plotting Cliff’s deltas of multiple experiments (Figure 6C). This differential was also observed for the RPA32/ssDNA PLA signal (Figure 6D). Notably however, in this case the mutant STING cells were comparable to the STING-deficient control rather than measuring below it (compare Figures 6C, D). Thus, it is possible to speculate that STING S358A has a gain-of-function phenotype of reducing specifically the nascent DNA-bound RPA level.

Phospho-RPA32 (S33P) measurements largely mirrored the above results. That is, in the STING S358A cells the RPA32 S33P/EdU level (by PLA, Figures 6E, F) but not the total nuclear RPA32 S33P (by IF, Figure 6G) was below the STING-deficient control. At the same time, the STING WT cells consistently displayed more of both RPA32 S33P/EdU and total RPA32 S33P than the STING mutant cells (Figures 6E–G). Interestingly however, relative to the STING-deficient control, the RPA32 S33P/EdU signal was highly variable in the STING WT cells (Figures 6E, F, 4G). One potential explanation may be an increased instability of phospho-RPA association with EdU-positive nascent DNA in the STING WT cells.

The above-mentioned differences between STING WT and S358A cell populations observed on a cell-by-cell basis were not as pronounced when assessed by probing the fractionated cell extracts with RPA and RPA S33P antibodies in Western blots, though we could discern some excess of RPA in NE/chromatin pellet fractions of STING WT over STING S358A cells (Supplementary Figure S5). This limited detection is not surprising given the differential abundance of RPA on the chromatin of S phase versus non-S phase cells (Supplementary Figure S4E). The state of RPA S33P in pellet fractions was not possible to assess due to the interference from non-specific bands (Supplementary Figure S5A).

**FIGURE 7:**
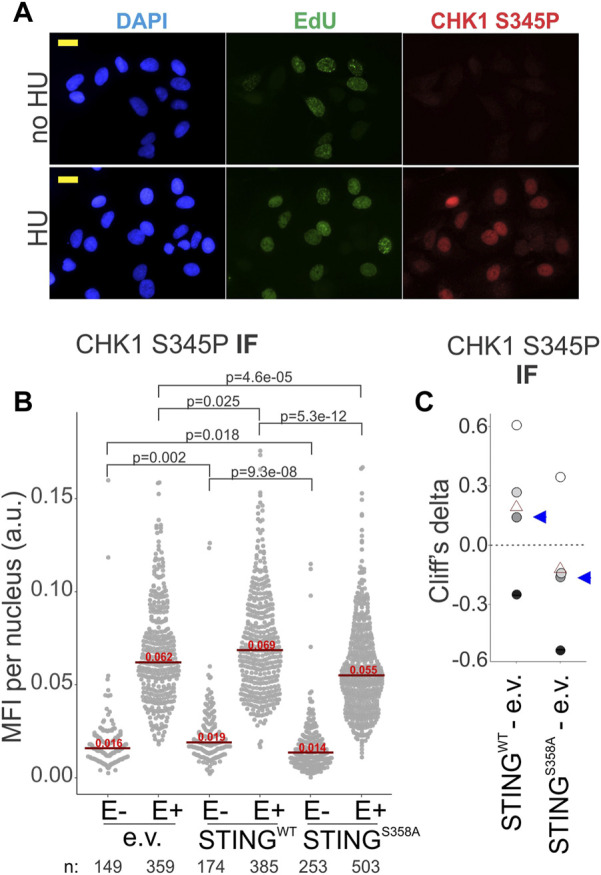
CHK1 activation is affected by STING status. **(A)** A representative image of CHK1 S345P and EdU immunofluorescence in HU-treated (5 mM HU/5 h) and untreated U2OS expressing wild type STING. Cells were treated as in Figure 6A. MFI, mean fluorescence intensity, a.u, arbitrary units. Scale bar = 20 μm. **(B)** IF images of CHK1 S345P were generated for the HU-treated U2OS cells with the indicated transgenes, and CHK1 S345P signal intensity was quantified. Signal intensities are shown separately for EdU-negative (E−) and EdU-positive (E+) cells. Red lines are medians and their values are shown above the lines. *p* values were determined in KS tests. **(C)** Cliff’s delta values for MFI distribution differences between the indicated pairs of samples were calculated for two independent experiments with biological replicates (i.e., four sets total) quantified as in **(A)**. Values derived for each set are identifiable by fill tone. Blue arrowheads identify the values from the experiment shown in **(A)**. *p* values are determined in KS tests.

At stalled forks, ssDNA-bound RPA is key to the activation of the ATR--CHK1 axis of the replication checkpoint (Bhat and Cortez, 2018). If in the STING S358A cells the ssDNA-bound RPA is less abundant compared to the STING WT cells, we should expect an effect on the ATR-CHK1 checkpoint induction. We performed IF *in situ* for the activated CHK1, i.e., CHK1 S345P, to address this (Figure 7A). Indeed, the STING S358A U2OS cells consistently showed lower levels of CHK1 S345P signal than the STING WT cells (Figures 7B, C). This tendency was also discernible on Western blots of the fractionated STING WT and STING S358A U2OS, where the latter cells displayed moderately less phospho-CHK1 and CHK1 in LS and chromatin/NE fractions (Supplementary Figure S5).

**FIGURE 8:**
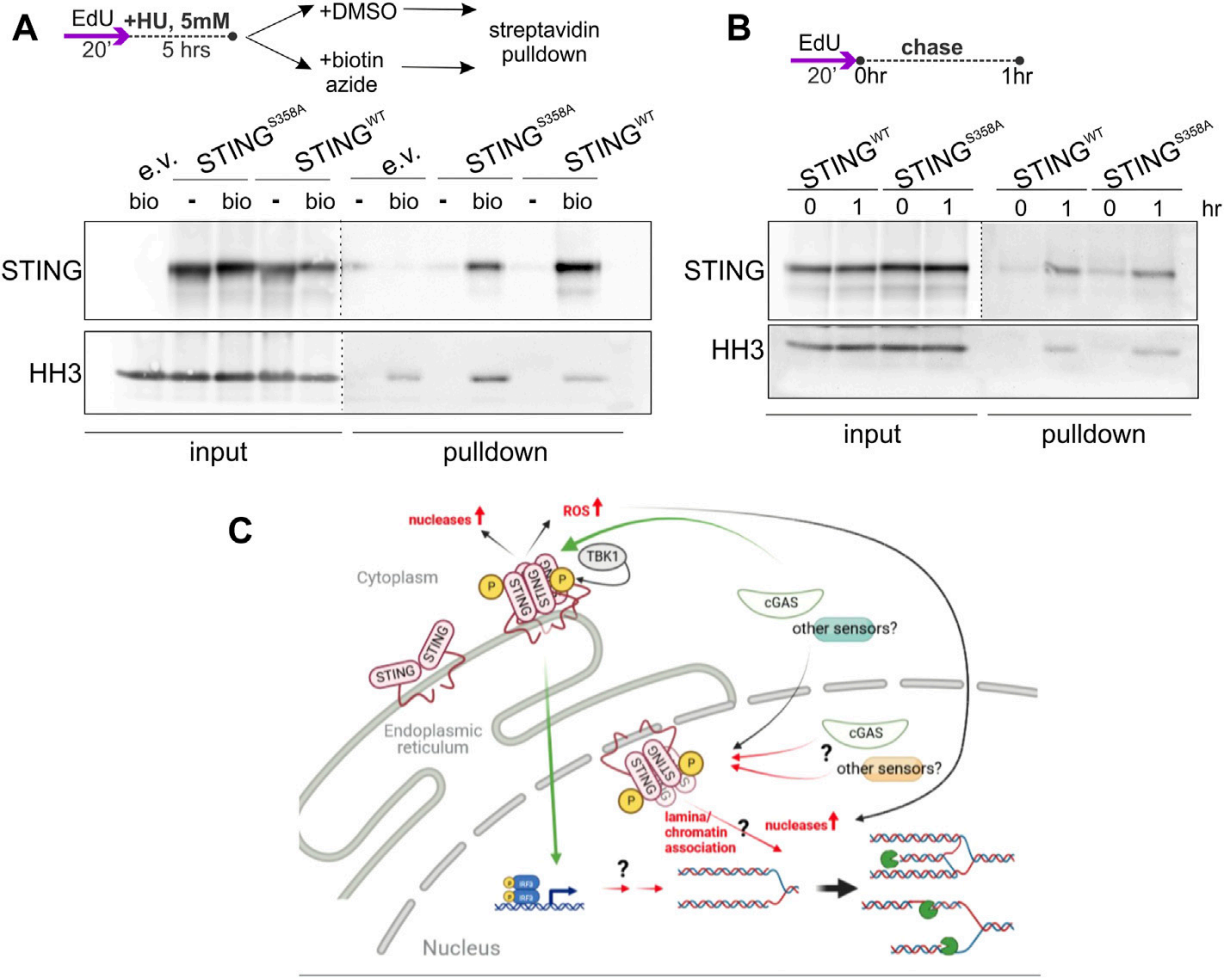
STING associates with chromatin in an immunoprecipitation of nascent DNA (iPOND) assay. **(A)** A schematic of EdU labeling, HU treatment, and sample preparation, and a Western blot of iPOND input and pulldown samples isolated from U2OS with the indicated transgenes or empty vector (e.v.), and probed with the STING and histone H3 (HH3) antibodies. Samples clicked with biotin azide: bio; mock-clicked samples: **-**. Dotted lines indicate different brightness settings and/or exposures of a Western blot image. **(B)** An EdU-labeling scheme and a Western blot of iPOND of samples of the same cells as in **(A)**, probed for STING and histone H3. **(A,B)** represent two independent experiments each. **(C)** A model for STING effect on nascent strand degradation, NDD. A canonical pathway *via* the ER-bound, activated STING to the transcriptional upregulation and/or ROS-mediated responses is a possibility that has not been ruled out. However, our data are consistent with the existence of an inner nuclear membrane pool of STING that is activatable and can participate in the regulation of fork metabolism *via* association with lamina and chromatin, or, similarly to the ER STING, that facilitates upregulation of the activity and/or levels of nucleases. Green arrows indicate canonical mechanisms. Black arrows indicate the documented relationships that may be drawn upon to explain STING’s effect on NDD. Red arrows are the proposed novel mechanisms. Pacman symbols indicate nucleolytic processing of stalled forks. The model was generated with BioRender.

While STING still can, in principle, exert its effect on NDD indirectly, detection of STING-dependent ssDNA/RPA responses, and of STING presence in the chromatin/NE fractions (Figures 1, 4, Supplementary Figures S1, S3) together with the Dixon et al. (2021) report of the inner nuclear membrane (INM) pool of STING, argue for nuclear involvement of STING. To further test this, we performed immunoprecipitation of nascent DNA (iPOND) on our U2OS cells, and detected both the wild type and mutant STING on the chromatin of HU-arrested forks (Figure 8A). Both versions of STING were also detectable at low level on unperturbed forks (Figure 8B), and interestingly, at a higher level on EdU pulse-labeled chromatin 1hr after fork passage (Figure 8B). Thus, while STING does not appear to be a fork-bound protein, it is associated with the maturing chromatin after replication and with the chromatin of the arrested forks that is known to undergo at least some steps of maturation (Sirbu et al., 2013; Kehrli et al., 2016). The S358A mutation clearly does not abolish association of STING with chromatin, as is also suggested by our fractionation studies, but a closer follow up in the future may reveal quantitative differences.

Overall, the results of this section suggest a potential replication fork-specific mediator of STING’s effect on NDD—the RPA protein—and place STING in physical proximity of arrested forks. In addition, the results establish a link between the replication checkpoint and innate immune responses to replication stress.

## Discussion

### Replication phenotypes of STING

In this work we showed that activation of STING during an HU-mediated replication fork arrest contributes to upregulation of nascent DNA degradation (NDD) at arrested forks in several different cell lines. These data complement the reports of the cGAS/STING activation in response to fork degradation (Emam et al., 2022) but are distinct in that they suggest that STING is not only downstream of the signal but can influence the signal’s generation. In addition, we showed that depletion of STING slowed fork progression rate in unperturbed cells in three different cell lines. However, re-expression of the protein increased fork rate in A549 but not nearly as much in U2OS.

Compared to the phenotype of increased fork rate, the NDD phenotype of arrested forks was more dependent on STING’s potential to activate. Specifically, overactivation of STING by an agonist consistently produced the latter phenotype but did not always produce the former. Second, an activation-incompetent STING S358A did not upregulate NDD but exhibited a partial fork rate increase in A549. These data may suggest that STING’s effect on unperturbed forks is contextual, depending on the steady state levels of STING itself or its regulators, or on the baseline level of signaling through the STING cascade.

The phenotype of reconstitution of STING in A549, whereby both the unperturbed fork rate and the stalled fork degradation increase, is virtually identical to the fork behavior in cells with disrupted Okazaki fragment processing, OFP (Thakar et al., 2020), and is thought to involve increased ssDNA gaps. As a novel determinant of NDD, OFP was proposed to be distinct from the canonical, BRCA2-and Fanconi Anemia pathway-dependent fork protection (FP) pathway (Thakar et al., 2020; Cong et al., 2021; Thakar and Moldovan, 2021). Based on our observations that the unperturbed and the arrested fork phenotypes of STING are not always linked and are not epistatic with BRCA1, we propose that STING may modify both FP and OFP. For example, it may affect the level of RPA-bound single stranded DNA at internal gaps behind a fork and/or at paired nascent strands of a reversed fork.

Specifically, we showed that STING’s effect on fork processing in U2OS is associated with changes in the levels of RPA on parental and nascent DNA. When bound to the parental ssDNA at forks, RPA can promote stalled fork reversal, which is a prerequisite for NDD (Bhat and Cortez, 2018; Berti et al., 2020), while nascent ssDNA-bound RPA participates in resection of this DNA (Soniat et al., 2019; Duan and Pathania, 2020). Therefore, detecting more RPA on parental and nascent ssDNA in the wild type STING-expressing cells is consistent with their elevated NDD. On the other hand, less RPA at ssDNA in the STING S358A cells and a greater reduction of RPA specifically on the nascent DNA may suggest that fork reversal is reduced, and the nascent ssDNA is less abundant or is unavailable for RPA binding and thus is protected from degradation. This nascent DNA phenotype appears to be a gain of function by STING S358A over STING deficiency. It can be proposed that the stronger STING S358A phenotype in reducing the RPA/nascent ssDNA versus the RPA/total ssDNA is consistent with its stronger reversal of NDD versus its partial reversal of the unperturbed fork rate phenotype of the wild type STING, because nascent ssDNA-bound RPA is likely more prevalent at arrested forks.

The replication phenotypes of STING described in this study do not mandate that STING associates with forks directly or even resides in the nucleus. For example, one hypothetical scenario of indirect action of STING on NDD is that STING activation induces ROS (Hayman et al., 2021), and ROS may activate the MRE11 nuclease (Somyajit et al., 2021) (see the model in Figure 8C). Also, upregulation of the cytoplasmic nuclease TREX1 has been reported downstream of active STING (Sooreshjani et al., 2018), and TREX1 was found to enter the nucleus during HU treatment in an early study (Yang et al., 2007). However, our data together with published reports converge on the notion that STING is not just an ER but also a nuclear actor. A subset of STING resides in the INM (Schirmer et al., 2003; Malik et al., 2014; Dixon et al., 2021) and associates with nuclear proteins (Cheradame et al., 2021; Dixon et al., 2021) and with chromatin (our work); it can also be activated at the INM (Dixon et al., 2021). The existence of the INM-localized, chromatin-interacting STING is further supported by the notion of a dynamic ER contiguous with both membranes of the NE, where protein targeting to the INM is determined by diffusion and retention *via* interactions with the nuclear lamina and chromatin (Ungricht and Kutay, 2015; De Magistris and Antonin, 2018). Indeed, Dixon et al. found lamins A/C and B1, the lamin-associated protein LAP2α, histones, and chromatin regulators among STING-interacting proteins. Malik et al. (2014) observed an effect of STING on chromatin compaction. We propose that the INM STING associates with chromatin in the context of lamina/chromatin complexes. This context provides ample opportunities for STING to affect NDD, since lamins and lamin-associated proteins are extensively implicated in the regulation of replication fork metabolism [reviewed in (Graziano et al., 2018; Willaume et al., 2021)], including but not limited to the recruitment of RPA (Graziano et al., 2021; Bao et al., 2022) (Figure 8C).

### Involvement of cGAS

Our data argue that cGAS is involved in activating STING under conditions of replication arrest, though it is possible that cGAS is not the only activator of STING in this context. A recently-identified non-canonical cGAS-independent, ATM/p53-dependent, DNA damage-responsive pathway (Dunphy et al., 2018), may also be engaged during replication arrest, as long as ATM is activated, and in fact we have observed ATM activation in the HU-treated cGAS KO HeLa (not shown). However, our data indicate that the contribution of the non-canonical pathway is not critical to the effect of STING on stalled replication forks, since we observe this effect both in p53-mutant (UWB1.289) and wild type (U2OS, A549, and HeLa) lines, and this effect is not detectable in the absence of cGAS.

Another important question regarding cGAS is whether it has an effect on forks in and of itself. The suppression of fork progression and NDD by cGAS (Chen et al., 2020) is so far considered to be STING-independent, and independent of the cGAS role as a STING activator. Mechanistically, it is not fully characterized. Our data indicate that at least in the context of a carcinoma-derived cell line, HeLa, knocking out cGAS does not induce a marked NDD as observed by Chen et al. (2020) in BJ primary fibroblasts. Quite possibly, this differential requirement for cGAS in fork protection may reflect cancer cells’ adaptive evolution, and/or depend on the degree of activation of the cGAS/STING pathway.

### Implications of the findings

Since the excised nascent DNA at stalled forks can be a trigger for the cGAS/STING activation (Bhattacharya et al., 2017; Coquel et al., 2018; Emam et al., 2022), our data imply existence of a positive feedback from the activated STING to fork processing. Such feedback should boost the response though not make it unlimited since the cGAS/STING pathway has built-in negative feedback loops (Hertzog and Rehwinkel, 2020; Hong et al., 2021). While at present such positive feedback remains speculative, it is no longer inconceivable. Recent work suggests that the immunostimulatory DNA sensor IFI16 also suppresses DNA repair in such a way as to increase the amount of this DNA in the cytoplasm (Ka et al., 2021).

Both the protection of nascent DNA and the cGAS/STING pathway are often suppressed in cancer (Sun et al., 2013; Xia et al., 2016; Deschamps and Kalamvoki, 2017; de Queiroz et al., 2018). Unprotected nascent DNA may reflect the reality of chronic replication stress in cancer cells, whereby fork stalling is a regular occurrence and genomic stability is no longer selected for. The cGAS/STING pathway inactivation may provide a benefit of dampening the inflammatory responses to the chronically elevated presence of fragments of genomic DNA due to replication stress and, related to it, aneuploidy and chromosomal instability. Our data suggest a further connection between fork protection and the cGAS/STING pathway: that the loss of the cGAS/STING pathway, in addition to its other adaptive advantages, can also benefit cancer cells by severing a link that amplifies NDD and checkpoint activation. Our observations that overactivation of the endogenous STING by an agonist can lead to a small but significant increase in NDD over the already high level due to the BRCA1-deficiency in an ovarian cancer cell line UWB1.289, as well as to a comparatively larger increase in the BRCA1-proficient SKOV-3, are consistent with this notion. In contrast, in normal cells where NDD is minimal, STING activation may be beneficial as it boosts the replication checkpoint signal from rare events of fork stalling. Greater mechanistic understanding of the STING effects on replication may therefore yield new targets for future therapeutic manipulation.

## Materials and methods

### Cells and culture

The SV40-transformed human fibroblast GM639 (GM00639, Cellosaurus ID CVCL_7299) has been used by us and others previously (Swanson et al., 2004; Sidorova et al., 2008) and was obtained from the NIGMS Human Genetic Mutant Cell Repository. Exome sequencing of this cell line was reported in (Juarez et al., 2018). The U2OS line was acquired from ATCC (ATCC HTB-96). UWB1.289 (DelloRusso et al., 2007) and SKOV-3 were a gift of Drs. Welcsh and Swisher. A549 was a gift of Drs. Tang and Monnat. WI38hTERT fibroblast line was a gift of Dr. Carl Mann (Jeanblanc et al., 2012) and also contains an inducible GFP-RAF1-ER transgene, however its expression was not induced under the conditions used in this study. GM639, U2OS, and A549 were grown in high glucose Dulbecco Modified Minimal Essential Medium (DMEM) with L-glutamine, 10% fetal bovine serum, FBS, (Hyclone) and antibiotics. UW289.B1 was grown in 1:1 mixture of RPMI and MGEM (Lonza) with Single Quots (Lonza) and 3% FBS. SKOV-3 was grown in McCoy’s 5A media supplemented with 10% FBS and antibiotics. The HeLa cGAS KO and complemented lines are a gift of Dr. Dan Stetson and were described in (Volkman et al., 2019). These cells were grown in high glucose DMEM with L-glutamine supplemented with 10% FBS and antibiotics. WI-38hTERT was grown in Minimal Essential Medium (MEM) with L-glutamine and sodium pyruvate (Gibco) supplemented with 10% FBS, Nonessential Amino Acids, and antibiotics. All cell lines were kept in a humidified 5% CO_2_, 37°C incubator. *Mycoplasma* testing was performed regularly using the UW/FHCRC Cancer Consortium Shared Resource Specimen processing service https://sharedresources.fredhutch.org/services/mycoplasma-testing.

### Drugs and other reagents

Stock of 5-iododeoxyuridine (IdU, Sigma-Aldrich) was at 2.5 mM in PBS, 5-chlorodeoxyuridine (CldU, Sigma-Aldrich) was at 10 mM in PBS, and 5-ethynyldeoxyuridine (EdU, Sigma-Aldrich or Click Chemistry Tools) was at 10 mM in DMSO. IdU and CldU were used at a concentration of 50 μM and EdU was used at 10 or 20 μM. Hydroxyurea (Sigma-Aldrich) stock solution was at 1M in PBS and mirin (Calbiochem) was at 10 mM in DMSO. Doxycycline stock solution was at 100 μg/ml in PBS and SR-717 (MedChemExpress) was at 10 mM in DMSO. All stocks were stored at −20°C.

### RNAi-mediated depletion

siRNAs against STING Hs_TMEM173_1 Cat. No. SI04132170 (target sequence 5′CCG​CAC​GGA​TTT​CTC​TTG​AGA3′) and Hs_TMEM173_4, Cat. No. SI04357696 (target sequence 5′CCG​GAT​TCG​AAC​TTA​CAA​TCA3′) and a Negative Control non-targeting siRNA were from Qiagen and transfected with lipofectamine RNAiMAX (Invitrogen) according to the manufacturer’s protocol. Experiments were performed 36–48 h post-transfection with individual siRNAs, which produced similar results. Depletion was verified in each transfection by Western blotting.

### Constructs

pTRIP-SFFV-mtagBFP-2A STING and the parental empty vector were a gift from Nicolas Manel (respectively, Addgene plasmid # 102586; http://n2t.net/addgene:102586; RRID: Addgene_102586; and Addgene plasmid # 102585; http://n2t.net/addgene:102585; RRID:Addgene_102585). The S358A mutation was introduced into the STING ORF in this construct using Q5 site-directed mutagenesis kit (NEB). Virus generation from these constructs and cell transduction were as described (Sidorova et al., 2008). Live transduced cells were sorted on the Aria flow sorter based on the level of BFP expression.

### Antibodies

Antibodies were as follows: Mouse α-biotin Cat. No. MB-9100 (Vector Laboratories); rat α-BrdU/CldU Cat. No. ab6326 (Abcam); mouse α-BrdU/IdU Cat. No.347580 (BD Biosciences); rabbit α-STING Cat. No.19851-1-AP (Proteintech), mouse α-NCL Cat. No. 396400 (Life Technologies), rabbit α-RPA32 Cat. No. A300-244A (Bethyl Labs), rabbit α-RPA32 S33P Cat. No. A300-246A (Bethyl Labs), rabbit CHK1 S345P Cat. No. 2348 (CST), mouse α-ssDNA MAB3034 (Millipore Sigma), rabbit α-cGAS Cat. No. 15102S (CST), mouse α-γH2AX clone JBW301, Cat. No. 05–636 (Millipore Sigma), rabbit α-DNA-PKcs Cat. No. 38168 (CST), mouse α-IFI16 Cat. No. sc-8023 (Santa Cruz Biotechnology), rabbit α-MRE11 Cat. No. NB100-142 (Novus biologicals), mouse α-LaminA/C Cat. No. sc-376248 (Santa Cruz Biotechnology), rabbit α-GAPDH Cat. No. 5174 (CST), rabbit α-Histone H3 Cat. No. 4499 (CST), rabbit α-FANCD2 Cat. No. A302-174A (Bethyl Labs), rabbit α-STING S366P Cat. No. 50907 (CST), rabbit α-ERp72 Cat. No. 5033 (CST), rabbit α-EMC1 Cat. No. A305-605A (Bethyl Labs), mouse α-WRN, Cat. No. W0393 (Millipore-Sigma).

Proteins were visualized on Western blots by ECL (Thermo Scientific) and quantified using FluorChem Imager (Alpha Inotech). For presentation, images were saved in TIFF format, adjusted for brightness/contrast and cropped in GIMP, then assembled into figures in CorelDraw. Image brightness/contrast adjustments were made across all lanes of each protein measured. In some cases, lane order was changed and extra lanes were deleted.

### Microfluidics assisted replication track analysis (maRTA)

This procedure was done as described (Sidorova et al., 2009; Kehrli and Sidorova, 2014). Microscopy of stretched DNAs was performed on the Zeiss Axiovert microscope with a ×40 objective, and images were captured with the Zeiss AxioCam HRm camera. Lengths of tracks were measured in raw merged images using Zeiss AxioVision software. Fluorochromes were Alexa594 for CldU, Alexa488 for IdU, and Neutravidin Texas Red for EdU.

### Transfection of interferon-stimulating DNA (ISD)

Two microgram of ISD (Invivogen) or 1 μg each of ISD and poly-dI/dC (Millipore-Sigma) was transfected into cells using lipofectamine 2000 (Invitrogen) per manufacturer’s protocol. Cells were incubated for 6 h and harvested for RNA analyses or fractionation. Mock-transfected controls received lipofectamine/Optimem mixture only.

### RNA isolation and qPCR

RNAs were isolated using RNeasy Plus RNA isolation kit (Qiagen). Two microgram of RNA was reverse-transcribed using High Capacity cDNA Reverse Transcription kit (Applied Biosystems) per manufacturer’s protocol. cDNAs were diluted 1:10 and 1 μl of diluted cDNA was used per qPCR reaction with iTaq Universal SYBR Green supermix (Bio-Rad) and the following pairs of primers: 5′ACA​ACT​TTG​GCA​TTG​AA3′ and 5′GAT​GCA​GGG​ATG​ATG​TTC​TG3′ for GAPDH; and Sigma-Aldrich KiCqStart predesigned pairs H_IFNB1_1 and H_IL6_1, Cat. No. KSPQ12012G for IFNb and IL6. Triplicate Ct values were averaged, normalized to GAPDH, and fold induction of mRNAs was determined according to the 2^−ΔΔCT^ method.

### Proximity ligation assay (PLA) and immunofluorescence (IF) *in situ*

PLA was performed using DuoLink red detection kit and DuoLink anti-mouse and anti-rabbit antibodies (Millipore-Sigma Cat. Nos. DUO92008, DUO92001, and DUO92002, respectively) as described previously (Lazarchuk et al., 2019), except that after formaldehyde fixation cells were washed in PBS, permeabilized by addition of 4°C 90% methanol in PBS and stored at −20°C prior to staining. Detergent extraction *in situ* prior to fixation and IF to visualize chromatin-bound RPA32 was performed according to (Mirzoeva and Petrini, 2003). Images of cells were collected under Zeiss Axiovert 200M microscope with ×40 magnification objective using Micro Manager software. Digital images were analyzed with Fiji ImageJ software package with custom macros as described in (Lazarchuk et al., 2019) or with Cell Profiler software package.

### Cellular fractionation

Fractionation was performed according to (Federation et al., 2020) with minor modifications. Nuclei isolation was done by incubating with .2% NP40 in the isolation buffer for 15 min. Isotonic wash prior to extraction of nuclei with Low Salt buffer was omitted. Insoluble pellets after High Salt buffer extraction were solubilized by resuspending in LDS sample buffer (Invitrogen), boiling and sonicating in Bioruptor Pico (Diagenode) or by resuspending in lysis buffer (50 mM HEPES-KOH pH7.5, 140 mM NaCl, 1 mM EDTA pH8.0, 1% Triton X-100, .1% Sodium Deoxycholate, .1% SDS, protease inhibitors) and sonicating. Fractionations in which phosphorylation of proteins was assessed included phosphatase inhibitors (Thermo Scientific) in all buffers.

### Immunoprecipitation of nascent DNA (iPOND)

iPOND was performed as in (Kehrli et al., 2016) on 1 × 10^7^ cells per sample, with the following modification: pulldowns were washed for 5 min each in SDS buffer (1% SDS in 50 mM Tris HCl pH 8.0), low salt buffer (1% Triton X-100, 20 mM Tris HCl pH 8.0, 2 mM EDTA, 150 mM NaCl, high salt buffer (1% Triton X-100, 20 mM Tris HCl pH 8.0, 2 mM EDTA, 500 mM NaCl), and lithium chloride wash buffer (100 mM Tris HCl pH 8.0, 500 mM LiCl, 1% NP40); then rinsed in PBS and boiled in 2xLDS buffer (Invitrogen) for loading on a PAGE.

## Statistical analysis

Statistical analyses and graphing of the data were done in R studio. *p* values for qPCR results were derived from pairwise t-tests on ΔΔCq values with Benjamini-Hochberg adjustment for multiple comparisons. *p* values for the rest of the assays were as follows. For continuous variables (e.g., track lengths, mean fluorescence intensities) *p* values were calculated in K.S. tests and for discrete variables (e.g. PLA foci)—in Wilcoxon tests, and the analyses were always performed on whole datasets, without exclusion of any outlier data points. To quantify and concisely visualize the sizes of differences between PLA foci, track lengths, or MFI distributions of pairs of samples we calculated the Cliff’s delta statistic for these pairs. Cliff’s delta is a metric recommended for comparison of non-parametric distributions. In general, Cliff’s delta of a distribution A vs. distribution B can range from 1 (if all values in A are larger than all values in B) to −1 (if the reverse is true), and 0 value indicates that the distributions are completely overlapping.

## Data Availability

The datasets presented in this article are not readily available because no datasets listed under point 2 were generated. Requests to access the datasets should be directed to julias@uw.edu.
