# Supplementary material for: Innate immunity mediator STING modulates nascent DNA metabolism at stalled forks in human cells
Source: Front Mol Biosci. 2023 Jan 12;9:1048726. doi: 10.3389/fmolb.2022.1048726 (PMC9877313; doi:10.3389/fmolb.2022.1048726)
Supplement: Supplementary file 1 [file Table1.DOCX]

**Supplementary Figures**

**
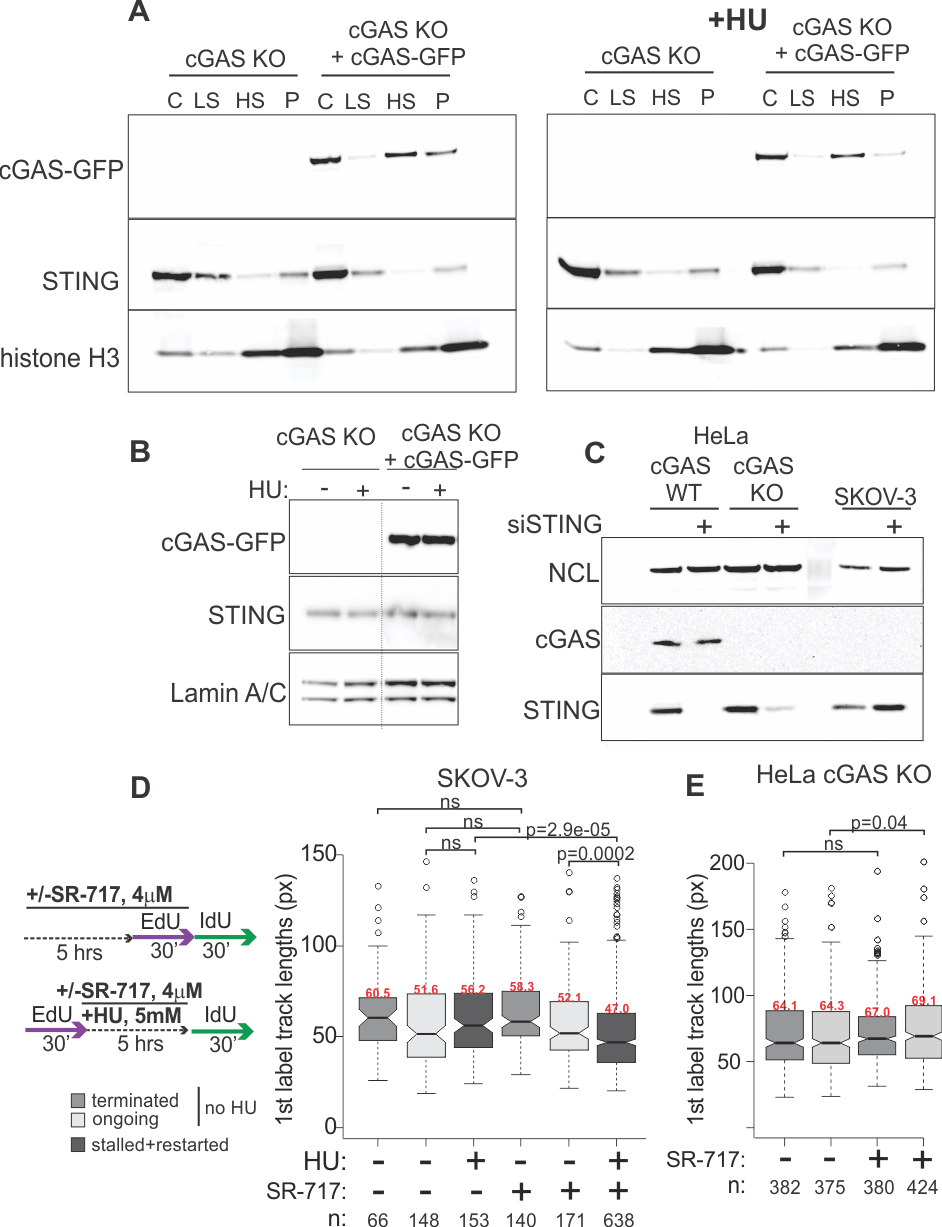
**

**Supplementary Figure 1**. A-B) Western blots of extracts of HeLa cGAS KO cells and their derivative that expresses cGAS-GFP were fractionated as described in Fig.1 and probed for the indicated proteins. All cells were treated with doxycycline for 20-24 hrs prior to fractionation. HU treatment (right panel) was for 5hrs at 5mM prior to fractionation. (A) shows all four fractions (P, chromatin pellet, LS, low salt extract, HS, high salt extract, C, cytoplasm) and (B) shows pellet fractions from a separate experiment. A dotted line marks a spliced-out lane. (A) and (B) represent three independent experiments. C) A Western blot comparing cGAS and STING levels in HeLa, HeLa cGAS KO, and SKOV-3 cells. siRNA against STING was transfected into cells where indicated. D) An experimental design for (D) and (E) and a boxplot comparing EdU track length distributions in SKOV-3 cells treated with SR-717 with or without HU treatment. E) A boxplot of EdU track length distributions in HeLa cGAS KO cells treated or not treated with SR-717 as in (D). P values in (D) and (E) were determined in KS tests and median values are shown in red above the boxplots. Px, pixels.

**
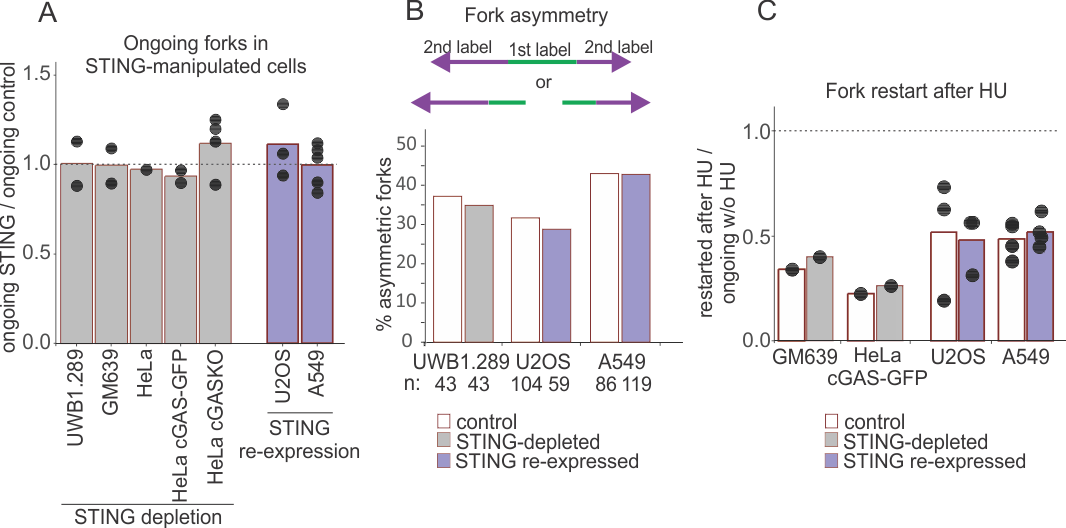
**

**Supplementary Figure 2.** Ongoing fork stability is unaffected by manipulation of STING levels in cells. A) For the indicated cell lines, relative proportion of ongoing forks within a sum of ongoing and terminated forks was calculated as a metric of the stability of ongoing forks in unperturbed S phase samples from multiple independent experiments. To compare across independent experiments, the values calculated for cells with altered STING levels (depleted or re-expressed) were normalized to those of the cognate controls. B) Fork asymmetry was determined by measuring the lengths of 2^nd^ label tracks in forks diverging from a single replication origin (identified as three-segment tracks or two two-segment tracks facing away from each other and separated by a small gap, as shown on the schematic). Diverging forks were considered asymmetric if their 2^nd^ label track lengths differed by more than 33%. Since diverging forks are relatively sparce, 2^nd^ label ratios for diverging forks from three (U2OS) and two (A549) independent experiments were combined. For UWB1.289, the results are from one independent experiment. C) Efficiency of fork restart after HU was calculated as a fold difference in relative abundances of restarted forks over those of ongoing forks in multiple independent experiments. In (A) and (C) dots represent independent experiments and bars are means.

**
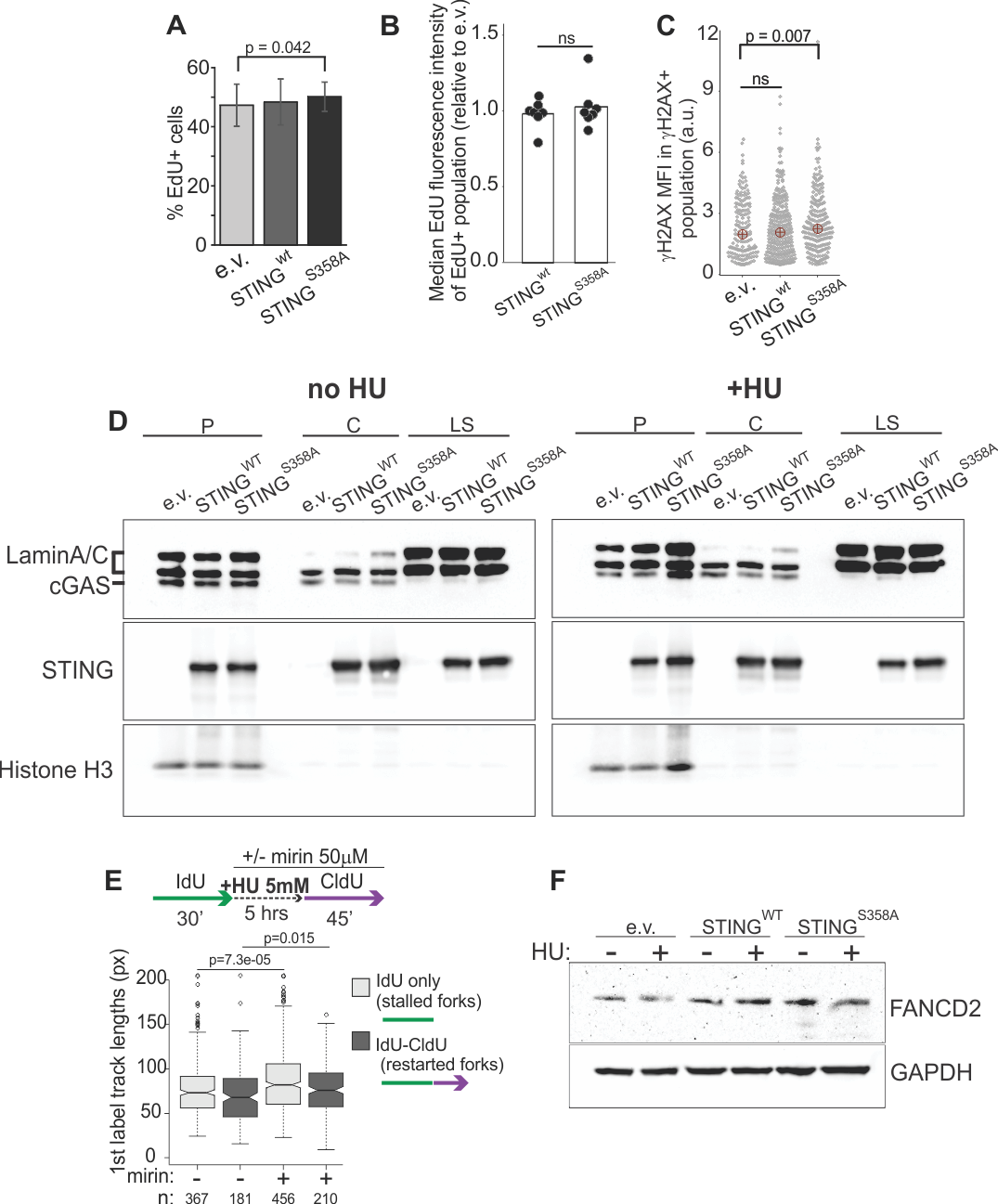
**

**Supplementary Figure 3.** Characterization of STING expression in U2OS cells. A) Percentage of EdU-positive cells in the untreated U2OS expressing the indicated transgenes. Cells were labeled with EdU for 20 min and analyzed by IF in situ. The results are averages of six independent experiments, and the p value was determined in a two-tailed paired t-test. Error bars are standard deviations. B) Median EdU incorporation levels in EdU-positive cells determined in the experiments shown in (A). These levels were normalized to the levels seen in U2OS with empty vector in order to compare across independent IF experiments. Bars represent the means, and significance was determined in a two-tailed t-test. C) γH2AX levels in γH2AX-positive subpopulations after treatment of U2OS expressing the indicated transgenes with 5mM HU for 5hrs. The graph represents two independent experiments with two biological replicates each. Red circles are means, and p values are determined in KS tests. D) Western blots of U2OS expressing vector control or the wild type or mutant STING. Cells were fractionated as in Figs.1 and S1. In the right panel cells were treated with 5mM HU for 5 hrs prior to fractionation. The blots represent two independent experiments. P, pellet, LS, low salt extract, C, cytoplasm. E) An experimental design and a boxplot of 1^st^ label (IdU) track length distributions in terminated and restarting forks from U2OS expressing STING and treated or not treated with 50uM mirin during HU arrest and recovery. p values are determined in KS tests. F) A Western blot of whole cell extracts of U2OS expressing vector control or the wild type or mutant STING probed for FANCD2 levels after unperturbed growth or 5-hr arrest with 5mM HU.

**
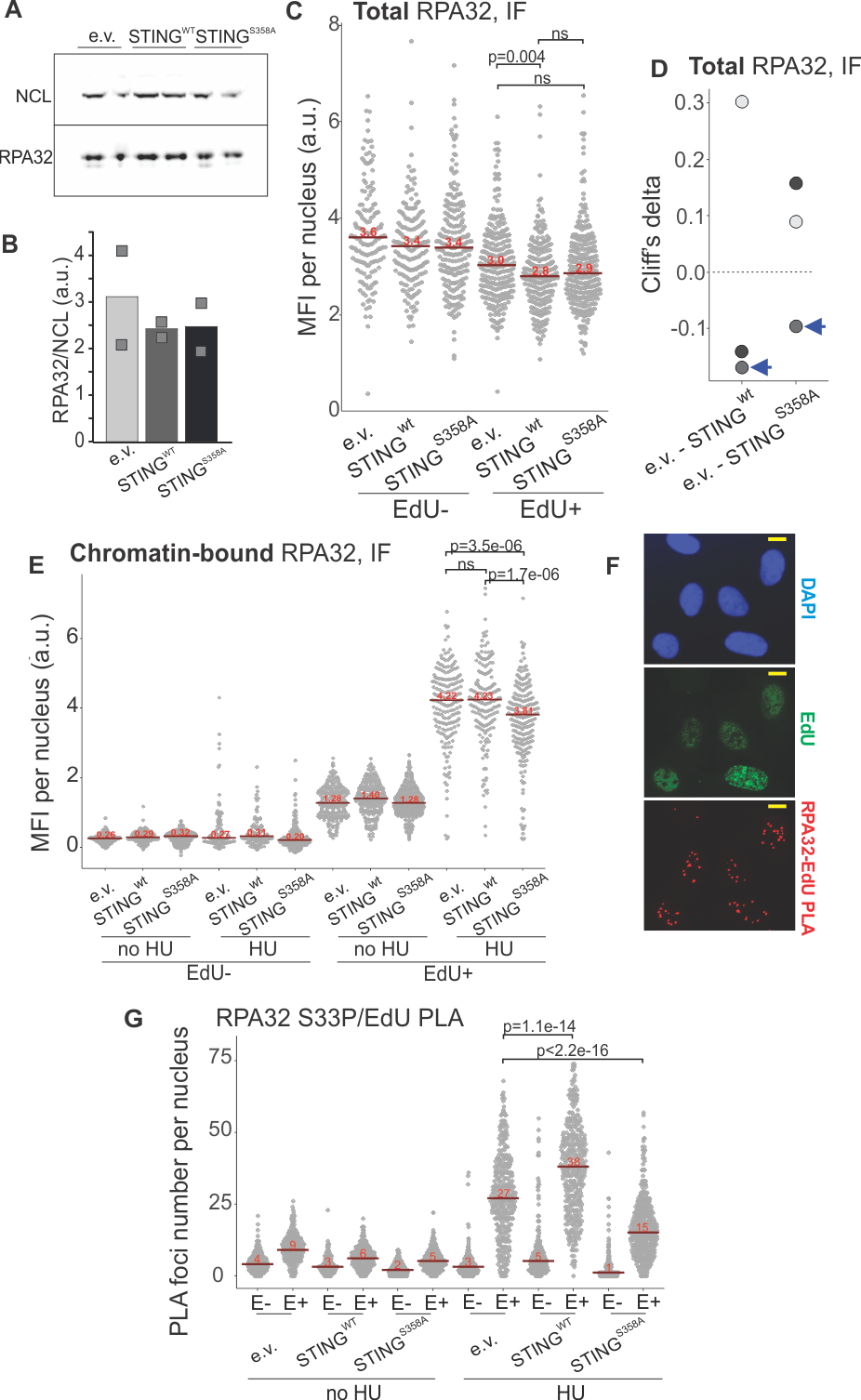
**

**Supplementary Figure 4.** Total and chromatin-bound RPA levels in U2OS. A, B) A Western blot (A) and a quantification of Western blot results (B) of RPA32 measurements in two biological replicates of U2OS cells expressing the empty vector (e.v.) or the indicated transgenes. Bars indicate means. C) A quantitation of IF in situ measurements of total nuclear RPA32 in the U2OS cells with the indicated transgenes or the empty vector. Data are presented separately for EdU-positive and EdU-negative cells. Cells were labeled with EdU for 20 min, treated with 5mM HU for 5 hrs, and fixed for IF. Red bars are medians and their values are shown above the bars. D) Cliff’s delta values derived from two independent experiments (one with two biological replicates) performed and analyzed as in (C). Blue arrowheads indicate the values obtained for the experiment presented in (C). E) A quantitation of IF in situ measurements of chromatin-bound RPA32 in the U2OS cells with the indicated transgenes or the empty vector. Data are presented separately for EdU-positive and EdU-negative cells. Cells were labeled with EdU for 20 min and treated or not treated with HU as above. Prior to fixation, cells were detergent-extracted in situ as described in Materials and Methods. Red bars are medians and their values are shown above the bars. In (C) and (E), p values were determined in KS tests. F) A split channel view of the RPA32 to EdU PLA image shown in Figure 6A. Scale bar=10μm. G) Distributions of RPA32 S33P/EdU PLA foci numbers in the U2OS cells with empty vector or the indicated STING transgenes. Cells were labeled with EdU and harvested immediately or after 5hrs of 5mM HU arrest. PLA foci numbers are shown separately for EdU-negative (E-) and EdU-positive (E+) cells. Red lines are medians and their values are shown above the lines. P values were calculated in Wilcoxon tests.


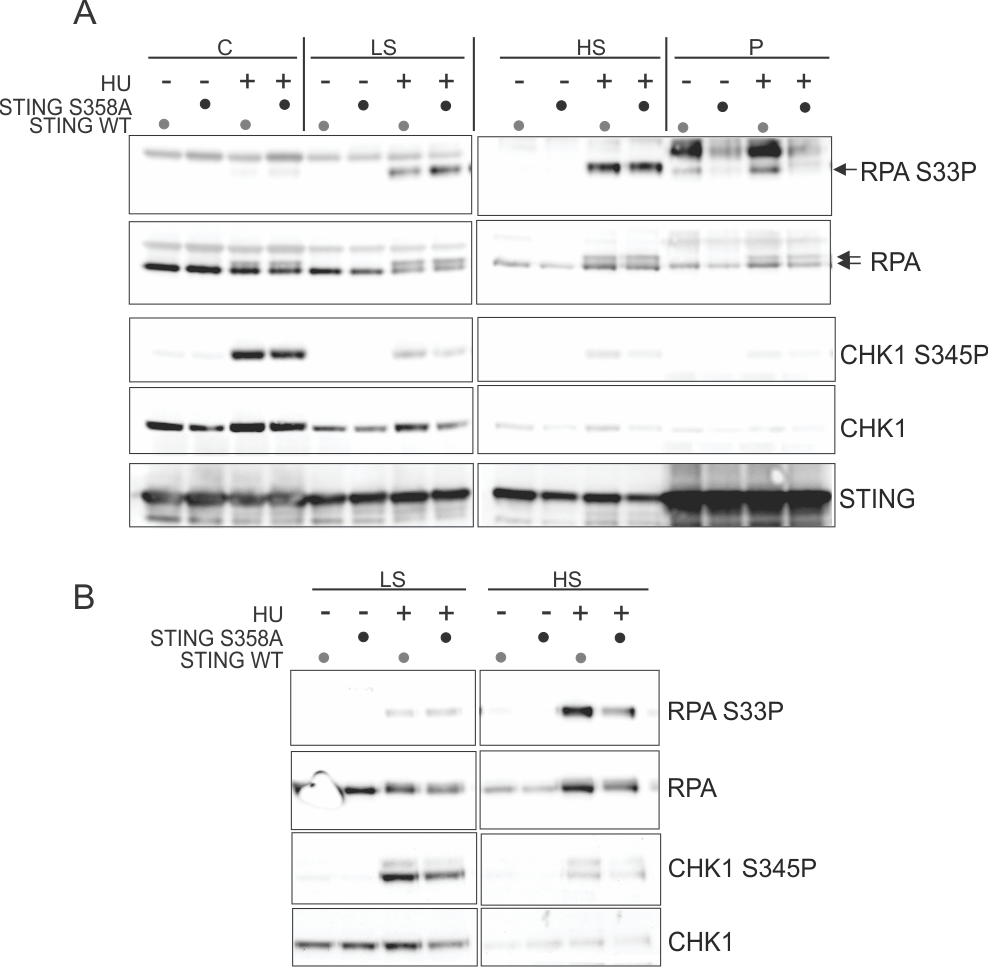


**Supplementary Figure 5**. Total levels of RPA, CHK1, and their phosphorylated forms in U2OS. (A) and (B) are independent experiments in which U2OS cells expressing STING WT or S358A mutant were fractionated as in Figs. 1, S1, and S3, and the fractions were probed on Western blots for the indicated proteins. HU treatment was at 5mM for 5-6 hrs. P, pellet, LS, low salt extract, HS, high salt extract, C, cytoplasm. Note that the bands bound by the RPA S33P antibody in pellet fractions are non-specific.
